# Efficacy of antihypertensive treatment for target organ protection in patients with masked hypertension (ANTI-MASK): a multicentre, double-blind, placebo-controlled trial

**DOI:** 10.1016/j.eclinm.2024.102736

**Published:** 2024-07-18

**Authors:** Jian-Feng Huang, Dong-Yan Zhang, De-Wei An, Ming-Xuan Li, Chang-Yuan Liu, Ying-Qing Feng, Qi-Dong Zheng, Xin Chen, Jan A. Staessen, Ji-Guang Wang, Yan Li, ANTI-MASK Investigators, Yan Li, Jian-Feng Huang, Dong-Yan Zhang, De-Wei An, Ming-Xuan Li, Yi-Qing Zhang, Ji-Guang Wang, Xin Chen, Chang-Yuan Liu, Gui-Li Chang, Zhe Hu, Ying-Qing Feng, Xi-Da Li, Can Liu, Jia-Yi Huang, Yu-Ling Yu, Qi-Dong Zheng, Yi-Yun Wang, Xue-Ning Zhang, Jing Yu, Rui-Xin Ma, Heng-Xia Liu, Xiao-Ping Chen, Qing-Tao Meng, Zhi-Peng Zhang, Yu Dou, Mei-Yu Zhu, Wen-Juan Wang, Li-Li Zhu, Min Zhang, Yi-Nong Jiang, Yan Lu, Wei Yu, Xiao-Ling Xu, Qiu-Yan Dai, Yu-Feng Zhu, Hui-Jie Zhang, Yu Zhang, Jin-Shun Zhang, Pei-Li Bu, Ling-Xin Liu, Jian-Jun Mu, Jing-Tao Xu, Yue-Yuan Liao, Hao Guo, Xin-Yue Liang, Jan A. Staessen

**Affiliations:** a Department of Cardiovascular Medicine, Shanghai Institute of Hypertension, Shanghai Key Laboratory of Hypertension, National Research Centre for Translational Medicine, State Key Laboratory of Medical Genomics, Ruijin Hospital, Shanghai Jiatong University School of Medicine, Shanghai, China; b Non-Profit Research Association Alliance for the Promotion of Preventive Medicine, Mechelen, Belgium; c Department of Cardiology, Guangdong Provincial Peoples' Hospital, Guangzhou, Guangdong, China; d Department of Internal Medicine, Yuhuan 2nd Peoples' Hospital, Taizhou City, Zhejiang Province, China; e Biomedical Research Group, Faculty of Medicine, University of Leuven, Leuven, Belgium

**Keywords:** Masked hypertension, Antihypertensive treatment, Ambulatory blood pressure monitoring, Randomised clinical trial

## Abstract

**Background:**

Masked hypertension is associated with target organ damage (TOD) and adverse health outcomes, but whether antihypertensive treatment improves TOD in patients with masked hypertension is unproven.

**Methods:**

In this multicentre, randomised, double-blind, placebo-controlled trial at 15 Chinese hospitals, untreated outpatients aged 30–70 years with an office blood pressure (BP) of <140/<90 mm Hg and 24-h, daytime or nighttime ambulatory BP of ≥130/≥80, ≥135/≥85, or ≥120/≥70 mm Hg were enrolled. Patients had ≥1 sign of TOD: electrocardiographic left ventricular hypertrophy (LVH), brachial-ankle pulse wave velocity (baPWV) ≥1400 cm/s, or urinary albumin-to-creatinine ratio (ACR) ≥3.5 mg/mmol in women and ≥2.5 mg/mmol in men. Exclusion criteria included secondary hypertension, diabetic nephropathy, serum creatinine ≥176.8 μmol/L, and cardiovascular disease within 6 months of screening. After stratification for centre, sex and the presence of nighttime hypertension, eligible patients were randomly assigned (1:1) to receive antihypertensive treatment or placebo. Patients and investigators were masked to group assignment. Active treatment consisted of allisartan starting at 80 mg/day, to be increased to 160 mg/day at month 2, and to be combined with amlodipine 2.5 mg/day at month 4, if the ambulatory BP remained uncontrolled. Matching placebos were used likewise in the control group. The primary endpoint was the improvement of TOD, defined as normalisation of baPWV, ACR or LVH or a ≥20% reduction in baPWV or ACR over the 48-week follow-up. The intention-to-treat analysis included all randomised patients, the per-protocol analysis patients who fully adhered to the protocol, and the safety analysis all patients who received at least one dose of the study medication. This study is registered with ClinicalTrials.gov, NCT02893358.

**Findings:**

Between February 14, 2017, and October 31, 2020, 320 patients (43.1% women; mean age ± SD 53.7 ± 9.7 years) were enrolled. Baseline office and 24-h BP averaged 130 ± 6.0/81 ± 5.9 mm Hg and 136 ± 8.6/84 ± 6.1 mm Hg, and the prevalence of elevated baPWV, ACR and LVH were 97.5%, 12.5%, and 7.8%, respectively. The 24-h BP decreased on average (±SE) by 10.1 ± 0.9/6.4 ± 0.5 mm Hg in 153 patients on active treatment and by 1.3 ± 0.9/1.0 ± 0.5 mm Hg in 167 patients on placebo. Improvement of TOD occurred in 79 patients randomised to active treatment and in 49 patients on placebo: 51.6% (95% CI 43.7%, 59.5%) versus 29.3% (22.1, 36.5%; p < 0.0001). Per-protocol and subgroup analyses were confirmatory. Adverse events were generally mild and occurred in 38 (25.3%) and 43 (26.4%) patients randomised to active treatment and placebo, respectively (p = 0.83).

**Interpretation:**

Our results suggest that antihypertensive treatment improves TOD in patients with masked hypertension, highlighting the need of treatment. However, the long-term benefit in preventing cardiovascular complications still needs to be established.

**Funding:**

Salubris China.

**Research in context**

**Evidence before this study**

We searched PubMed (from January 1, 1970, to October 30, 2021), Embase (from January 1, 1970, to October 30, 2021) for relevant articles on March 10, 2024, using the search terms “masked hypertension”, “target organ damage” and “randomised controlled trial”. We could not identify any randomised controlled trial on masked hypertension. Previous observational studies have demonstrated that masked hypertension was associated with increased risk of cardiovascular mortality and morbidity, and patients with masked and sustained hypertension had similar levels of target organ damage. However, whether antihypertensive treatment can bring benefit to patients with masked hypertension remains unproven.

**Added value of this study**

Compared to placebo, antihypertensive treatment in patients with masked hypertension reduced office and ambulatory blood pressure and improved target organ damage as captured by the composite of electrocardiographic voltage criteria, brachial-ankle pulse wave velocity, and the urinary albumin-to-creatinine ratio.

**Implications of all the available evidence**

Patients with masked hypertension require antihypertensive treatment guided by out-of-office blood pressure monitoring. However, the long-term benefit of the treatment in preventing cardiovascular complications still needs to be established.

## Introduction

Population studies across all races and ethnicities highlight that hypertension is the major modifiable driver of cardiovascular complications and by far the leading risk factor causing death and disability.^1^, ^2^, ^3^ Current guidelines propose that the management of hypertension requires out-of-office blood pressure (BP) monitoring.^4^^,^^5^ Masked hypertension is a normal office BP, as measured by a physician or nurse in a medical environment, such as a general practice or an out-patient clinic, combined with an elevated out-of-office BP, recorded by ambulatory or home BP monitoring. Masked hypertension carries a risk similar to that associated with sustained hypertension, i.e., hypertension on office and out-of-office BP measurement.^6^ In the International Database on Ambulatory BP in Relation to Cardiovascular Outcome (IDACO),^1^ 14.6% of participants had masked hypertension and 25.5% had sustained hypertension. Compared to individuals with normotension, the multivariable-adjusted hazard ratios expressing the risk of experiencing a major cardiovascular endpoint amounted to 1.62 and 1.80 in patients with masked and sustained hypertension, respectively.^1^ Furthermore, in observational studies,^7^, ^8^, ^9^ patients with masked and sustained hypertension had similar levels of target organ damage (TOD).

Until now, no randomised clinical trial addressed the question whether antihypertensive treatment of masked hypertension entails benefit. We therefore mounted the double-blind placebo-controlled ANTI-MASK Trial (NCT02893358) to assess whether in patients with masked hypertension BP lowering treatment for 1 year would improve TOD, as captured by brachial-ankle pulse wave velocity (baPWV),^10^ the urinary albumin-to-creatinine ratio (ACR), and electrocardiographic left ventricular hypertrophy. Considering the moderate reproducibility of masked hypertension,^11^ the diagnosis was confirmed at two screening visits 1 month apart.

## Methods

### Study design

ANTI-MASK was a multicentre, randomised, double-blind, placebo-controlled clinical trial. We adhered to the principles of the Helsinki declaration. The study protocol, the statistical analysis plan, and protocol amendments are available in Appendix B. The Ethics Committee of Ruijin Hospital, Shanghai, China, approved the study protocol. Patient recruitment took place from February 14, 2017 until October 31, 2020, at 15 clinical sites in China (Table S1 in Appendix B). For the current report, we adhered to the reporting guidelines recommended by the CONSORT Group.^12^

### Participants

At each of two screening visits, 1 month apart, office and 24-h BP were measured. Patients of either sex, aged 30–70 years, with masked hypertension confirmed at two screening visits were eligible, provided that they had not been treated for hypertension or were off treatment for at least 2 weeks, had at least one sign of TOD and were willing to be followed up for 1 year. The exclusion criteria were suspected or confirmed secondary hypertension, liver dysfunction, serum creatinine concentration ≥176.8 μmol/L, diabetic nephropathy, renal parenchymal disease, peripheral arterial disease, severe cardiovascular and non-cardiovascular disease, mental illness, substance abuse, and participation in another study. Specific ANTI-MASK exclusion criteria included atrial fibrillation and arrhythmia, because of interference with oscillometric BP measurement, and known intolerance for angiotensin receptor blockers or dihydropyridine calcium-channel blockers. All participants provided informed written consent.

### Randomisation and masking

After stratification for centre, sex and the presence versus absence of nighttime hypertension,^13^^,^^14^ eligible patients were randomised in a 1:1 proportion to antihypertensive drugs or matching placebos, using a computerised random function and permuted blocks. Tablets and pill boxes of the active antihypertensive drugs and their matching placebos had identical appearance. Investigators, who enrolled and followed patients or assessed outcome measures, and patients were all masked to group assignment.

### Procedures

Office BP was the average of three consecutive oscillometric readings obtained with the patients resting seated by validated^15^ OMRON HBP1100 devices (Omron Healthcare Co., Ltd., Kyoto, Japan). At each screening visit, the 24-h ambulatory BP was recorded, using validated^16^ A&D TM-2430 monitors (A&D Company Ltd., Tokyo, Japan). The monitors were programmed to obtain readings at 20-min intervals during the day (6 AM–10 PM) and at 30-min intervals at night (10 PM–6 AM). For analysis, the ambulatory BP was averaged over 24 h and over the awake and asleep periods of the day as recorded in the patient diaries. Masked hypertension was an office BP of <140 mm Hg systolic and <90 mm Hg diastolic in the presence of ambulatory hypertension. The ambulatory hypertension thresholds applied were ≥130/≥80 mm Hg, ≥135/≥85 mm Hg, and ≥120/≥70 mm Hg for the 24-h, daytime and nighttime, respectively.^5^ When the ambulatory systolic or diastolic BP were in different categories, the highest category determined eligibility.

Fasting blood samples were collected by venipuncture and in each centre analysed by automated methods in certified laboratories. Signs of TOD included: electrocardiographic left ventricular hypertrophy (Sokolow-Lyon index ≥3.5 mV in women and ≥4.0 mV in men or with the calibration set at 10 mm a Cornell product ≥2440 mm × ms); ACR of ≥3.5 mg/mmol in women and ≥2.5 mg/mmol in men in two mid-morning urine samples collected on different days; and arterial stiffening defined as a baPWV of ≥1400 cm/s. After patients had rested in the supine position for ≥5 min, baPWV was measured using the Omron Vascular Profiler-1000/2000 device (Omron Healthcare Co., Ltd., Kyoto, Japan).^17^ The travel path was estimated from body height as the distance between the sternal notch and the ankle minus the distance between the sternal notch and brachial artery. baPWV was the travel path divided by the time difference between the foot of the oscillometrically detected brachial and tibial pulse waves (Fig. S1 in Appendix B). The left- and right-sided baPWV were averaged for analysis. Quality of life was scored in four domains, using the brief investigator-administered questionnaire developed by the World Health Organization (WHOQOL-BREF).^18^

Follow-up visits were scheduled at monthly intervals until month 4 and at 3-month intervals from month 6 to 12. Office BP was measured at each visit. The 24-h ambulatory BP monitoring was performed at 2 and 12 months, and additionally at 4 months for patients who had uncontrolled ambulatory BP at the 2-month follow-up. TOD was reassessed at months 3, 6, 9, and 12. The quality-of-life score was graded at baseline and at month 12. Active treatment was initiated with allisartan 80 mg once daily. If the 24-h, daytime or nighttime BP remained uncontrolled, the allisartan dose had to be doubled to 160 mg once daily from month 2 onwards and amlodipine 2.5 mg once daily had to be added at the 4-month visit. In the control group, matching placebos were used likewise.

### Outcomes

The primary endpoint was improvement of TOD, consisting of one or more of the following outcomes: a decrease in baPWV by ≥20% or to <1400 cm/s; an ACR reduction by ≥20% or to <3.5 mg/mmol in women and <2.5 mg/mmol in men; or normalisation of the Sokolow-Lyon index or the Cornell product. Secondary outcomes included changes in the 24-h, daytime and nighttime BPs at months 2 and 12, TOD at months 3, 6, 9, and 12, and the quality-of-life score at month 12. Monitoring of safety included recording of adverse events and checking routine blood and urinary tests at baseline and last follow-up.

### Statistical analysis

For data management and statistical analysis, SAS software, version 9.4 (SAS Institute, Cary, NC) was used. Sample size calculations assumed that given a dropout rate of 25%, improvement of TOD, the primary composite endpoint, would be reached in 40% of patients assigned to active treatment and in 20% of patients randomised to placebo.^19^ With power set at 0.90 and the 2-sided α-level at 0.05, the required sample size was 160 patients per group.

The Kolmogorov–Smirnov test was applied to assess the normality of distributions. The central tendency and spread of continuously distributed variables were presented as mean (SD) or median (interquartile range, IQR), as appropriate. Between-group means and proportions were compared by *t* test or Wilcoxon test, depending on the variable distribution, and Fisher exact test, respectively. Statistical significance was a two-tailed α-level of 0.05 or less.

The main analysis was implemented according to the intention-to-treat (ITT) principle and included all randomised patients. Two types of ITT analyses were performed according to the method of handling missing values. In the primary ITT analysis, missing values were substituted by multiple imputation^20^ based on a comprehensive conditional specification, including randomisation group, sex, age, and data at previous visits. The imputed data from 50 runs were pooled using the SAS MIANALYZE procedure. The secondary ITT analysis was based on observed data only, and serial measurements were analysed with the individual patients modelled as random effect, using the MIXED and GENMOD procedures, as implemented in the SAS package, for continuous and binary outcomes, respectively. The between-group differences in continuously distributed variables were obtained by subtracting the mean change in the placebo group from the corresponding change on active treatment. Within-group changes over multiple time points with baseline as reference were assessed by computing least square means from the MIXED and GENMOD models. Models accounted for the baseline level of the variable, sex and age as fixed effects and patient as random effect, and included randomisation group as class variable for the between-group comparisons. The 95% confidence interval (CI) of proportions was computed as *p^* ± 1.96 × √ (*p^* × [100—*p^*]/N), where *p^* and N are the proportion of individuals (range 0–100%) and the number of participants, respectively. The per-protocol analysis excluded patients, who did not adhere to the assigned treatment or who had violated the protocol. Similar to the secondary ITT analyses, the per-protocol analysis was based on observed data only.

### Role of the funding source

The funders had no role in study design, data collection, data analysis, data interpretation, or writing of the report. All authors had full access to the data in the study and had final responsibility for the decision to submit for publication.

## Results

### Patient enrolment

**Fig. 1:**
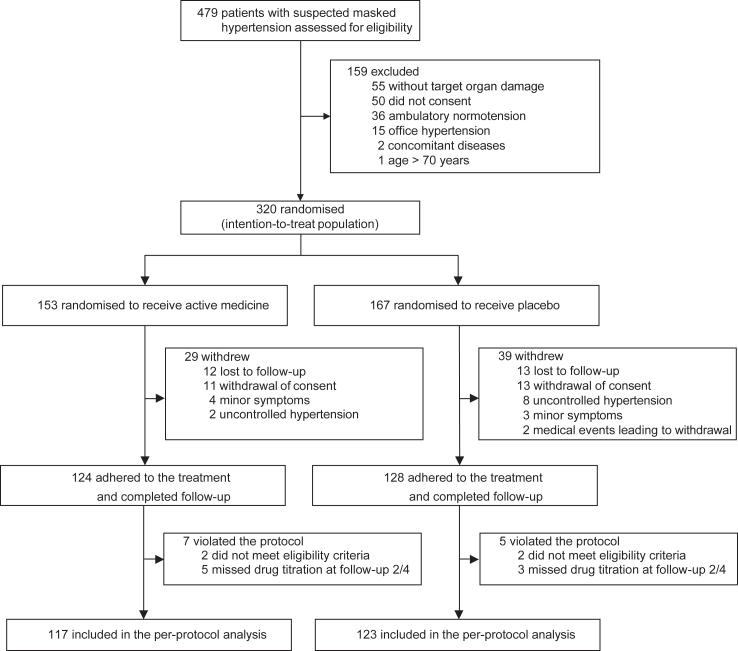
**CONSORTdiagram**. The chart shows patient disposition, including screening, randomisation and follow-up, and selection of patients for analysis.

From February 14, 2017, until October 31, 2020, 479 patients were screened for eligibility; 159 did not qualify, because they had office hypertension (n = 15), ambulatory normotension (n = 36), or did not have TOD (n = 55). One patient was older than 70 years, two had concomitant disease, and 50 declined participation. Finally, 320 patients were randomised: 153 to active treatment and 167 to placebo, and included in the ITT analysis (Fig. 1). Of the patients randomised to the active treatment, two did not meet all eligibility criteria and five missed the drug titration steps at month 2 or 4; of those randomised to placebo, these numbers were two and three, respectively (Fig. 1).

### Baseline characteristics

Patients randomised to active treatment and placebo had similar characteristics (Table 1). Among all 320 patients, 138 (43.1%) were women and 49 (15.3%) had been treated for hypertension. Office systolic/diastolic BP averaged (SD) 129.9 (6.0)/81.4 (5.9) mm Hg and the 24-h, daytime and nighttime BP 136.4 (8.6)/84.4 (6.1) mm Hg, 140.6 (9.5)/87.4 (6.8) mm Hg and 125.7 (10.5)/77.1 (6.6) mm Hg, respectively. Nighttime hypertension was present in 293 (91.6%) patients. The prevalence of 24-h, daytime and nighttime ambulatory hypertension ranged from 57.2% to 85.9% and from 51.3% to 86.6% at the first and second screening visits, respectively, with moderate concordance between the two visits (Table S2 in Appendix B).

**Table 1:** Baseline characteristics of participants.

Characteristic	Active treatment (n = 153)	Placebo (n = 167)
**No. with characteristics (%)**		
Women	64 (41.8)	74 (44.3)
Men	89 (58.2)	93 (55.7)
Current smoking	27 (17.7)	38 (22.8)
Drinking alcohol	32 (20.9)	39 (23.4)
Diabetes^a^	6 (3.9)	8 (4.8)
Use of antidiabetic drugs	5 (3.3)	2 (1.2)
Lipid-lowering treatment	14 (9.2)	18 (10.8)
Nighttime hypertension^b^	139 (90.9)	154 (92.2)
Previous antihypertensive treatment^c^	20 (13.1)	29 (17.4)
**Mean (SD) of measurements**		
Age, y	54 (9.4)	53 (10.1)
Body mass index, kg/m^2^^d^	24.4 (2.6)	24.3 (2.9)
Serum creatinine, μmol/L^e^	75.9 (15.6)	73.2 (15.3)
eGFR, mL/min/1.73 m^2^^e^^,^^f^	91.2 (12.8)	93.6 (12.9)
Total serum cholesterol, mmol/L^e^	5.26 (1.06)	5.12 (1.06)
HDL serum cholesterol, mmol/L^e^^,^^g^	1.32 (0.37)	1.30 (0.32)
Fasting plasma glucose, mmol/L^e^	5.31 (0.86)	5.38 (0.80)
**No. with target organ damage (%)**		
Left ventricular hypertrophy^h^	9 (5.9)	16 (9.6)
baPWV ≥1400 cm/s^i^	149 (97.4)	163 (97.6)
Microalbuminuria^j^	20 (13.1)	20 (12.0)

a Diabetes is a diagnosis documented in hospital records, using antidiabetic drugs or a fasting plasma glucose of ≥7 mmol/L.

b Nighttime hypertension is an ambulatory blood pressure of ≥120 mm Hg systolic or ≥70 mm Hg diastolic during sleep.

c Previous antihypertensive treatment was discontinued for ≥2 weeks prior to screening.

d Body mass index is body weight in kilogram divided by body height in meters squared.

e Conversion factors: creatinine from μmol/L to mg/dL, multiply by 0.0113; eGFR from mL/min/1.73 m^2^ to mL/s/1.73 m^2^, multiply 0.0167; cholesterol from mmol/L to mg/dL, multiply by 38.67; glucose from mmol/L to mg/dL, multiply by 18.02.

f eGFR is the glomerular filtration rate estimated from serum creatinine by the Chronic Kidney Disease Epidemiology Collaboration formula.

g HDL indicates high-density lipoprotein.

h Left ventricular hypertrophy is a Sokolow-Lyon index of ≥3.5 mV in women and ≥4.0 mV in men or a Cornell product ≥2440 mm × ms).

i baPWV indicates brachial-ankle pulse wave velocity.

j Microalbuminuria is a baseline urinary albumin-to-creatinine ratio (ACR) of ≥3.5 mg/mmol in women and ≥2.5 mg/mmol in men. Baseline level was the mean ACR of the two mid-morning urine samples obtained at the screening visits.

With regard to TOD, 25 patients (7.8%) had electrocardiographic left ventricular hypertrophy, 40 (12.5%) had microalbuminuria and 312 (97.5%) had baPWV ≥1400 cm/s, indicative of arterial stiffening. In all participants, mean values (SD) were 2.07 (0.69) mV for the Sokolow-Lyon index, 1468 (588) mm × ms for the Cornell product, and 1616 (191) cm/s for baPWV. The median urinary ACR (IQR) amounted to 1.55 (1.06–2.39) mg/mmol in women and 1.02 (0.63–1.48) mg/mmol in men. Compared to patients randomised in the trial, consenting patients who were not randomised had broadly similar characteristics with the exception of higher office diastolic BP, lower 24-h systolic BP, lower fasting plasma glucose and lower prevalence of TOD (Table S3 in Appendix B).

### Intention-to-treat analysis

Over the 1-year follow-up, 124 of 153 patients (81.0%) randomised to active treatment and 128 of 167 patients (76.6%) allocated to placebo completed the trial. All patients completing follow-up were adherent to the randomised treatment without addition of open-label antihypertensive drugs. Considering the two baseline ambulatory recordings and the repeat recordings at 2, 4 and 12 months, the median number of ambulatory readings recorded ranged from 61 to 62, from 43 to 44, and from 15 to 16 for the 24-h, daytime and nighttime BP, respectively (Table S4 in Appendix B).

In the active treatment group (n = 124), 46 patients (37.1%) were on allisartan 80 mg per day, 26 (21.0%) on allisartan 160 mg per day, and 52 (41.9%) on allisartan 160 mg plus amlodipine 2.5 mg per day; the corresponding number of patients in the placebo group (n = 128) were 19 (14.9%), 9 (7.0%) and 100 (78.1%), respectively. Thus, compared with active treatment, patients randomised to control needed the higher allisartan placebo dose and the addition of the amlodipine placebo (p < 0.0001).

In the primary ITT analysis, from randomisation to last follow-up, the office and ambulatory BP decreased on active treatment, but not on placebo (Table 2). With adjustments applied for the baseline BP, sex and age, the between-group differences (active treatment minus placebo [95% CI]) in the changes of office and 24-h systolic/diastolic BP were −9.1 [−11.6, −6.6]/−5.4 [−7.1, −3.7] mm Hg and −8.8 [−11.3, −6.3]/−5.4 [−6.7, −4.0] mm Hg, respectively. The corresponding values for daytime and nighttime BP were −8.8 [−11.4, −6.1]/−5.1 [−6.6, −3.7] mm Hg and −8.5 [−11.2, −5.8]/−5.2 [−6.8, −3.6] mm Hg, respectively. On a continuous scale the TOD changes in the electrocardiographic voltages, baPWV and ACR are summarised in Table 3. Compared to placebo, active antihypertensive treatment significantly reduced the Sokolow-Lyon index (−0.13 [−0.23, −0.02] mV, p = 0.018), the Cornell product (−100.9 [−189.0 to −12.9] mm × ms, p = 0.025), baPWV (−125.1 [−166.0 to −84.2] cm/s, p < 0.0001) and ACR (−0.30 [−0.38 to −0.23] mg/mmol, p = 0.0025).

**Table 2:** Blood pressure in the primary intention-to-treat analysis.

Blood pressure	Active treatment (n = 153)	Placebo (n = 167)	Between-group difference	p
Office SBP, mm Hg				
Baseline	130.4 (5.4)	129.4 (6.4)	1.0 (−0.3 to 2.3）	
Adjusted changes	−8.8 (0.9)‡	0.3 (0.9)	−9.1 (−11.6 to −6.6)	<0.0001
Office DBP, mm Hg				
Baseline	81.7 (5.2)	81.2 (6.4)	0.5 (−0.8 to 1.7)	
Adjusted changes	−4.2 (0.6)‡	1.2 (0.6)∗	−5.4 (−7.1 to −3.7)	<0.0001
24-h SBP, mm Hg				
Baseline	136.2 (7.7)	136.5 (9.4)	−0.3 (−2.2 to 1.6)	
Adjusted changes	−10.1 (0.9)‡	−1.3 (0.9)	−8.8 (−11.3 to −6.3)	<0.0001
24-h DBP, mm Hg				
Baseline	84.5 (5.7)	84.4 (6.5)	0.1 (−1.3 to 1.4)	
Adjusted changes	−6.4 (0.5)‡	−1.0 (0.5)∗	−5.4 (−6.7 to −4.0)	<0.0001
Daytime SBP, mm Hg				
Baseline	140.2 (8.3)	140.9 (10.4)	−0.7 (−2.7 to 1.4)	
Adjusted changes	−10.2 (1.0)‡	−1.4 (0.9)	−8.8 (−11.4 to −6.1)	<0.0001
Daytime DBP, mm Hg				
Baseline	87.2 (6.2)	87.5 (7.3)	−0.3 (−1.8 to 1.2)	
Adjusted changes	−6.4 (0.5)‡	−1.2 (0.5)∗	−5.1 (−6.6 to −3.7)	<0.0001
Nighttime SBP, mm Hg				
Baseline	125.8 (10.9)	125.6 (10.3)	0.3 (−2.1 to 2.6)	
Adjusted changes	−9.4 (1.0)‡	−0.9 (0.9)	−8.5 (−11.2 to −5.8)	<0.0001
Nighttime DBP, mm Hg				
Baseline	77.5 (6.6)	76.7 (6.7)	0.8 (−0.6 to 2.3)	
Adjusted changes	−6.1 (0.6)‡	−0.9 (0.6)	−5.2 (−6.8 to −3.6)	<0.0001

Baseline values are mean (SD). Adjusted changes are mean (SE). Between-group differences are presented as mean (95% confidence interval). In the primary intention-to-treat analysis, missing values are substituted by multiple imputation (see Statistical Methods). Within-group changes with baseline as reference were assessed by computing least square means from the MIXED and GENMOD models. Models accounted for the baseline level of the variable, sex and age as fixed effects and patient as random effect, and included randomisation group as class variable for the between-group comparisons. Office and ambulatory blood pressure at baseline are averages of measurements obtained at the two screening visits. SBP and DBP indicate systolic and diastolic blood pressure. Daytime and nighttime are awake and asleep periods of the day as recorded in the patient diaries. Significance of the within-group change: ∗p ≤ 0.05; ‡p ≤ 0.001.

**Table 3:** Target organ damage in the primary intention-to-treat analysis.

Variables	Active treatment (n = 153)	Placebo (n = 167)	Between-group difference	p
Sokolow-Lyon index, mV				
Baseline	2.08 (0.65)	2.06 (0.73)	0.01 (−0.14 to 0.17)	
Adjusted changes	−0.12 (0.04)†	0.004 (0.04)	−0.13 (−0.23 to −0.02)	0.018
Cornell product, mm × ms				
Baseline	1416.4 (505.5)	1515.2 (652.8)	−98.8 (−228.1 to 30.4)	
Adjusted changes	−83.4 (31.0)†	17.5 (32.0)	−100.9 (−189.0 to −12.9)	0.025
baPWV, cm/s				
Baseline	1622.1 (180.9)	1610.0 (199.7)	12.1 (−30.1 to 54.3)	
Adjusted changes	−152.8 (14.8)‡	−27.7 (14.8)	−125.1 (−166.0 to −84.2)	<0.0001
ACR, mg/mmol				
Baseline	1.27 (0.79–1.97)	1.20 (0.67–1.90)	0.06 (−0.17 to 0.35)	
Adjusted changes	−0.18 (0.09)∗	0.16 (0.08)	−0.30 (−0.38 to −0.23)	0.0025

Baseline values are mean (SD) or geometric mean (interquartile range). In the primary intention-to-treat analysis, missing values are substituted by multiple imputation (see Statistical Methods). Mixed models include randomisation group as class variable and account for the baseline value of the variable, sex and age as fixed effects and patient as random effect. Between-group differences are presented as mean (95% confidence interval). baPWV indicates brachial-ankle pulse wave velocity and ACR the urinary albumin-to-creatinine ratio. p refers to the significance of the between-group difference. Significance of the within-group change: ∗p ≤ 0.05; †p ≤ 0.01; ‡p ≤ 0.001.

**Fig. 2:**
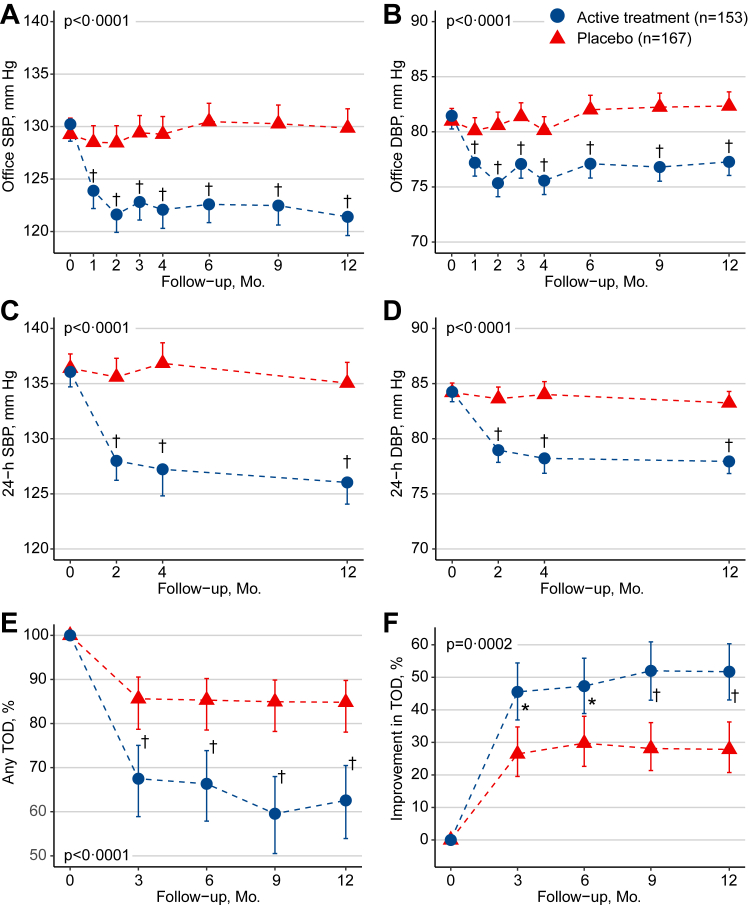
**Blood pressure and target organ damage in the primary intention-to-treat analysis**. The Figure shows the time trends by randomisation group for systolic and diastolic office blood pressure (panels A and B), the 24-h systolic and diastolic ambulatory blood pressure (panels C and D), the proportion of target organ damage (panel E), and the improvement proportion in target organ damage, the primary study outcome (panel F). SBP and DBP indicate systolic and diastolic blood pressure, and TOD target organ damage. Significance of the between-group differences: ∗p < 0.05; †p < 0.01; ‡p < 0.001.

On active treatment, from randomisation to last follow-up, the prevalence of left ventricular hypertrophy changed from 5.9% to 2.0% (p = 0.10), arterial stiffness from 97.4% to 60.3% (p < 0.0001) and microalbuminuria from 13.1% to 10.0% (p = 0.27). On placebo, the corresponding changes were from 9.6% to 8.0% (p = 0.56) for left ventricular hypertrophy, from 97.6% to 80.2% (p < 0.0001) for arterial stiffness and from 12.0% to 18.4% (p = 0.050) for microalbuminuria. Combining the three outcomes, the between-group difference was significant (p < 0.0001, Fig. 2, Panel E). Furthermore, active treatment reduced baPWV and ACR by ≥20% in 13.1% and 11.5% patients, compared with 3.6% and 5.8% on placebo (p = 0.0062 and p = 0.085 for the between-group differences, respectively). Overall, improvement of TOD (Fig. 2, Panel F), the primary outcome, occurred in 79 patients randomised to active treatment and in 49 randomised to placebo: 51.6% (95% CI 43.7%, 59.5%) versus 29.3% (22.1, 36.5%; p < 0.0001). Fig. S2 in Appendix B shows the changes over time in the three TOD components by treatment group. The between-group differences were significant for baPWV (p ≤ 0.0005), but not for the other signs of TOD (p ≥ 0.052). The secondary ITT analysis produced confirmatory results for BP and TOD analysed on a continuous scale and for the time trends in these outcomes (Table S5, Table S6 and Fig. S3 in Appendix B). There were no between-group differences in any of the four domains of quality of life (p ≥ 0.33; Table S7 in Appendix B).

Subgroup analyses were conducted in the framework of the primary ITT analysis and stratified by sex or the medians of age (55 years), body mass index (24 kg/m^2^), and 24-h systolic BP at randomisation (135 mm Hg), history of antihypertensive treatment prior to screening, and the presence versus absence of 24-h, daytime or nighttime hypertension (Fig. S4 in Appendix B). Among all strata, only the presence versus absence of daytime hypertension reached significance (p = 0.017) with greater TOD benefit in patients with daytime hypertension.

### Per-protocol analysis

The per-protocol analysis excluded 36 patients randomised to active treatment and 44 randomised to placebo, because patients withdrew or violated the protocol (Fig. 1, and Table S8 in Appendix B). The per-protocol analysis confirmed the BP changes observed in the ITT analysis as well as the changes in TOD (Tables S9 and S10, Fig. S5 in Appendix B).

### Safety analysis

The safety analysis excluded 3 patients randomised to active treatment and 4 randomised to placebo, who did not take any study medication. Adverse events were generally mild (Table S11 in Appendix B) and occurred in 38 (25.3%) and 43 (26.4%) patients randomised to active treatment and placebo, respectively (p = 0.83). Serious adverse events leading to hospitalisation occurred in 3 (2.0%) patients randomised to active treatment and 4 (2.5%) patients on placebo. No patient died during follow-up.

## Discussion

The key finding of the current study is that the initiation of antihypertensive drug treatment, compared with follow-up on placebo not only reduces the office and ambulatory BP, but also subclinical TOD, as captured by electrocardiographic voltage criteria signifying left ventricular hypertrophy, increased arterial stiffness as measured by baPWV, and microalbuminuria assessed by ACR.

As TOD is a forerunner of major cardiovascular complications, the clinical implications are obvious. In line with the unanimous recommendations in recent guidelines for the management of BP^4^^,^^5^ using out-of-office BP measurement, preferably by ambulatory monitoring,^21^ is indicated in all patients likely to have masked hypertension, irrespective of their treatment status. The increased risk of TOD in the presence of masked hypertension was first recognized in 1999, when it was reported that patients with masked hypertension had a higher left ventricular mass and more severe carotid atherosclerosis compared with truly normotensive individuals.^22^ Subsequent studies have demonstrated that masked hypertension was associated with increased cardiovascular mortality and morbidity. In a meta-analysis of 6 studies involving 30,352 patients who experienced 1327 events, the multivariable-adjusted hazard ratio of a composite cardiovascular event was 1.80 (95% Cl 1.57–2.06) for masked uncontrolled hypertension versus controlled hypertension.^23^ Subgroup meta-analysis showed that adjusted hazard ratio was 1.83 (1.52–2.21) in studies using ambulatory BP monitoring and 1.75 (1.38–2.20) in those using home BP measurement. Compared with normotensive people, patients with masked hypertension also have greater risk of developing sustained hypertension, i.e., office combined with ambulatory hypertension. In a Canadian population-based cohort, 37% of patients with masked hypertension at baseline developed sustained hypertension over 5 years of follow-up.^24^ In a Finnish study, this proportion was 73% over 11 years.^25^

In a longitudinal population-based cohort study of 11,135 adults from Europe, Asia, and South America, higher 24-h and nighttime BP were significantly associated with greater risks of death and a composite cardiovascular outcome, even after adjusting for other office-based or ambulatory BP measurements, such as dipping status or the night-to-day BP ratio.^14^ Thus, 24-h and nighttime BP may be considered optimal measurements for estimating cardiovascular risk. Moreover, isolated nocturnal hypertension has a prevalence of 7% among Whites and of 10%–11% among Blacks and Asians, and confers a risk higher than normotension.^13^^,^^26^^,^^27^ These observations justify why in the current study masked hypertension was defined as a normal office and an elevated ambulatory BP, irrespective of the period of the day, be it the 24-h, daytime or nighttime.

Masked hypertension is prevalent in populations and therefore should not be ignored in clinical practice.^8^^,^^9^^,^^28^ Its prevalence is highly dependent on the characteristics of participants under study. Indeed, the probability of having masked hypertension increases with an office BP in the high-normal range, age 40 years or older, overweight or obesity, excessive alcohol intake, diabetes mellitus, and smoking.^29^ In general population studies, the prevalence of masked hypertension is around 15%, while among hypertensive patients the prevalence varies with the BP range and the presence of comorbidities but averages 25%.^9^^,^^23^ In ANTI-MASK, at the first screening visit, patients with masked hypertension and likely to have TOD were invited to participate, explaining the prevalence of approximately 70% among screened patients.

ANTI-MASK is a randomised, double-blind placebo-controlled trial. In the current study, masked hypertension was confirmed based on office and ambulatory BP monitoring at two screening visits one month apart. Notwithstanding these strong points, several limitations should be considered. First, the primary outcome was a composite of signs indicating silent TOD. ANTI-MASK did not formally address the question whether in the long-term antihypertensive treatment would prevent transition of asymptomatic hypertensive TOD to major cardiovascular complications. Second, the observed regression of TOD was mainly driven by the normalisation or reduction of baPWV. As a marker of arterial stiffness, baPWV can predict cardiovascular outcomes independent of BP.^10^ However, it can also be reduced by vasodilators, even after a short-term treatment period. Third, in the current study left ventricular hypertrophy was based on electrocardiographic criteria with low sensitivity and specificity compared with more expensive and labour-intensive imaging approaches, in particular echocardiography. Future trials might embrace echocardiography rather than electrocardiography to capture left ventricular structure and function alterations. Fourth, in the subgroup analyses, the number of endpoints was small, so that the results from the stratified analysis should be considered as exploratory. The analysis contrasting sexes and patients with nighttime hypertension to those without were the only prespecified subgroup analyses, given that sex and nighttime hypertension were stratification factors prior to randomisation. Finally, our study population was confined to Chinese patients with confirmed masked hypertension and at least one sign of TOD. Therefore, our results need to be confirmed in patients of other ethnicities and in the prevention of TOD in patients with masked hypertension.

In conclusion, the double-blind placebo-controlled ANTI-MASK trial addressed the question whether antihypertensive treatment guided by ambulatory BP monitoring improves TOD in patients with masked hypertension. Compared to placebo, active treatment reduced office and ambulatory BP and improved TOD. The clinical implication is that in keeping with current guidelines^4^^,^^5^ out-of-office BP monitoring is required for the management of hypertension in patients with suspected masked hypertension and that in such patients the BP lowering treatment should be guided by the out-of-office BP.

## Contributors

JAS, JGW and YL contributed to the study design and the development of the protocol. JGW and YL obtained funding. JFH, DYZ, DWA, MXL, CYL, YQF, QDZ and XC contributed to the data acquisition. JFH, DYZ, DWA, JAS and YL did the statistical analysis. JFH, DYZ, DWA, MXL, JAS and YL drafted the manuscript. YQF, QDZ, XC, JGW and YL provided administrative, technical and material support. All authors had full access to the data in the study and had final responsibility for the decision to submit for publication. JFH, DWA, and YL have accessed to and verified the underlying study data.

## Data sharing statement

Anonymised individual participant data and a data dictionary defining each field in the dataset can be made available to investigators for targeted non-commercial research based on a motivated request to be submitted to the corresponding author. Proposals will be reviewed with scientific merit and feasibility as sole criteria. After approval of a proposal, data will be shared via a secure online platform after a data access and confidentiality agreement has been signed and after clearance has been obtained from the Ethics Committee of Ruijin Hospital, Shanghai, China. Data will be made available for a maximum of up to 2 years after sharing the requested data.

## Declaration of interests

JGW reports having received research grants, lecture and consulting fees from A&D, Bayer, Novartis, Omron, Servier and Viatris. YL reports having received research grants from A&D, Bayer, Omron, Salubris, and Shyndec and lecture fees from A&D, Omron, Servier, Salubris and Shyndec. All other authors declare no competing interests.
