# Supplementary material for: Efficacy of antihypertensive treatment for target organ protection in patients with masked hypertension (ANTI-MASK): a multicentre, double-blind, placebo-controlled trial
Source: eClinicalMedicine. 2024 Jul 18;74:102736. doi: 10.1016/j.eclinm.2024.102736 (PMC11293515; doi:10.1016/j.eclinm.2024.102736)
Supplement: Appendix A [file mmc1.doc]

**eClinicalMedicine**

**Appendix A**

The following translations were submitted by the authors and we reproduce them as supplied. They have not been peer reviewed. Our editorial processes have only been applied to the original abstract in English, which should serve as reference for this manuscript.

此份简体中文翻译由作者提交，未经同行评审。eClinicalMedicine的编辑流程仅适用于英文原稿，该原稿应作为本摘要的参考依据。

Supplement to: Huang JF, Zhang DY, An DW, Li MX et al. “Efficacy of antihypertensive treatment for target organ protection in patients with masked hypertension (ANTI-MASK): a multicentre, double-blind, placebo-controlled trial”

**中文摘要**

**背景**

隐蔽性高血压与靶器官损伤（TOD）和不良健康结局相关，但抗高血压治疗是否会改善隐蔽性高血压患者的 TOD 尚未得到证实。

**方法**

本研究在 15 家中国医院进行，为一项多中心、随机、双盲、安慰剂对照试验。入选年龄 30-70 岁、未服用抗高血压药物、诊室血压 <140/<90 mm Hg 且 24 小时、或白天或夜间动态血压分别≥130/ ≥80、≥135/≥85 或≥120/≥70 mm Hg的门诊患者。患者有≥1种TOD：心电图判断的左心室肥厚（LVH）、臂踝脉搏波传导速度（baPWV）≥1400 cm/s，或尿白蛋白肌酐比（ACR）女性≥3.5 mg/mmol，男性≥2.5 mg/mmol。排除标准包括继发性高血压、糖尿病肾病、血肌酐≥176·8 µmol/L，以及筛选6个月内发生的心血管疾病等。按照中心、性别和是否存在夜间高血压进行分层，符合入组标准的患者1：1随机分组为抗高血压治疗或安慰剂治疗。患者和研究者都不知晓分组情况。抗高血压治疗组先使用阿利沙坦酯80mg/天，若动态血压在治疗 2 个月后仍未得到控制则增加到 160 mg/天，治疗4 个月动态血压仍未控制则加用苯磺酸氨氯地平 2.5 mg/天。安慰剂对照组使用同样的加量方法。主要观察终点为治疗48周后的TOD改善率，定义为baPWV、ACR 或 LVH 正常化，或 baPWV 或 ACR 降低 ≥20%。意向性治疗分析包括进入随机的所有患者，符合方案集分析包括完全依从方案的患者，安全性分析包括服用了至少一次试验药物的所有患者。这个研究在ClinicalTrials.gov的注册号为NCT02893358.

**结果**

从2017年2月14日到2020年10月31日， 320名患者（43·1%女性，平均年龄±SD 53·7 ± 9·7岁）被随机入组。基线诊室血压平均为 130 ± 6·0 /81 ± 5·9 mm Hg，24 小时血压平均为136 ± 8·6 /84 ± 6·1 mm Hg。基线baPWV升高、ACR升高 和 LVH的患病率分别为 97·5%、12·5% 和 7·8%。降压药物治疗组153 名患者的24小时收缩压/舒张压平均（± SE）降低10·1 ± 0·9 /6·4 ± 0·5 mm Hg，安慰剂治疗组167名患者平均降低1·3 ± 0·9/1·0 ± 0·5 mmHg。降压药物治疗组和安慰剂组分别有79例和49例患者 TOD 改善，改善率（95%可信区间）分别为51·6% (43·7%, 59·5%) 及29·3% (22·1, 36·5%)，两组相比 p<0·0001。该结果在符合方案集及亚组分析中均得到证实。试验中发生的不良事件都比较轻微，降压药物治疗组和安慰剂组分别有38例（25·3%）和43例（26·4%）患者发生不良事件，两组相比 p=0·83。

**结论**

研究结果显示抗高血压治疗可改善隐蔽性高血压患者的 TOD，提示抗高血压治疗的必要性。然而, 抗高血压治疗预防隐蔽性高血压患者发生心血管并发症的长期获益仍需要证实。
