# Supplementary material for: Efficacy of antihypertensive treatment for target organ protection in patients with masked hypertension (ANTI-MASK): a multicentre, double-blind, placebo-controlled trial
Source: eClinicalMedicine. 2024 Jul 18;74:102736. doi: 10.1016/j.eclinm.2024.102736 (PMC11293515; doi:10.1016/j.eclinm.2024.102736)
Supplement: Appendix B [file mmc2.doc]

**eClinicalMedicine**

**Appendix B: Supplementary data and Study Protocol**

This Data Supplement formed part of the original submission and has been peer reviewed.
Supplement to: “*Efficacy of antihypertensive treatment for target organ protection in patients with masked hypertension (ANTI-MASK): a multicentre, double-blind, placebo-controlled trial”*

Huang JF, Zhang DY, An DW, Li MX, Liu CY, Feng YQ, Zheng QD, Chen X, Staessen JA, Wang JG, Li Y, for the ANTI-MASK Investigators.

**Table of contents Page**

**Table S1** ANTI-MASK trial centres and investigators 2

**Table S2** Ambulatory blood pressure thresholds indicative of masked hypertension 3

**Table S3** Comparison of patients randomised and not randomised 4

**Table S4** Number of ambulatory blood pressure readings 5

**Table S5** Blood pressure in the secondary intention-to-treat analysis 6

**Table S6** Target organ damage in the secondary intention-to-treat analysis 7

**Table S7** Quality-of-life score in the primary intention-to-treat analysis 8

**Table S8** Patients not included in the per-protocol analysis 9

**Table S9** Blood pressure in the per-protocol analysis 10

**Table S10** Target organ damage in the per-protocol analysis 11

**Table S11** Safety analysis by treatment group 12

**Figure S1** Measurement of brachial-ankle pulse wave velocity 13

**Figure S2** Individual TOD component in the primary intention-to-treat analysis 14

**Figure S3** Blood pressure and target organ damage in the secondary
intention-to-treat analysis 15

**Figure S4** Improvement of target organ damage by subgroups 16

**Figure S5** Blood pressure and target organ damage in the per-protocol analysis 17

**Study Protocol** 18-52

**Table S1:** **ANTI-MASK** **trial centres and investigators**

| **Centres** | **City** | **Investigators** | **ITT/PP** |  |
| --- | --- | --- | --- | --- |
| Ruijin Hospital Affiliated to Shanghai Jiaotong University School of Medicine | Shanghai | Yan Li, Jian-Feng Huang,  Dong-Yan Zhang, De-Wei An, Ming-Xuan Li, Yi-Qing Zhang, Ji-Guang Wang | 150/119 |  |
| Ruijin Hospital North Branch Affiliated to Shanghai Jiaotong University School of Medicine | Shanghai | Xin Chen, Chang-Yuan Liu,  Gui-Li Chang, Zhe Hu | 50/40 |  |
| Department of Cardiology, Guangdong Provincial Peoples’ Hospital | Guangzhou | Ying-Qing Feng, Xi-Da Li,  Can Liu, Jia-Yi Huang,  Yu-Ling Yu | 40/26 |  |
| Department of Internal Medicine, Yuhuan 2nd Peoples’ Hospital | Taizhou | Qi-Dong Zheng, Yi-Yun Wang, Xue-Ning Zhang | 30/23 |  |
| Department of Cardiology, 2^nd^ Hospital of Lanzhou University | Lanzhou | Jing Yu, Rui-Xin Ma,  Heng-Xia Liu | 10/8 |  |
| West China Hospital, Sichuan University | Chengdu | Xiao-Ping Chen, Qing-Tao Meng, Zhi-Peng Zhang | 8/5 |  |
| Jiangsu Province Geriatric Hospital | Nanjing | Yu Dou, Mei-Yu Zhu | 8/2 |  |
| Third Xiangya Hospital of Central South University | Changsha | Wen-Juan Wang, Li-Li Zhu,  Min Zhang | 7/5 |  |
| The First Affiliated Hospital of Dalian Medical University | Dalian | Yi-Nong Jiang, Yan Lu | 6/6 | |
| Zhejiang Hospital, Zhejiang University School of Medicine | Hangzhou | Wei Yu, Xiao-Ling Xu | 3/3 | |
| Shanghai General Hospital, Shanghai Jiaotong University School of Medicine | Shanghai | Qiu-Yan Dai, Yu-Feng Zhu | 2/2 | |
| Shenzhen Sun Yat-Sen Cardiovascular Hospital | Shenzhen | Hui-Jie Zhang, Yu Zhang,  Jin-Shun Zhang | 2/0 | |
| Qilu Hospital of Shandong University | Jinan | Pei-Li Bu, Ling-Xin Liu | 2/0 | |
| The First Affiliated Hospital of Xi’an Jiaotong University | Xi’an | Jian-Jun Mu, Jing-Tao Xu,  Yue-Yuan Liao | 1/1 | |
| The First Affiliated Hospital of Kunming Medical University | Kunming | Hao Guo, Xin-Yue Liang | 1/0 | |

ITT/PP refers to the number of patients included in the intention-to-treat (ITT) and per-protocol (PP) analysis.

**Table S2:** **Ambulatory blood pressure thresholds indicative of masked hypertension**

| **Blood pressure level** |  | **Screening visits** | | |
| --- | --- | --- | --- | --- |
|  |  | **First** | **Repeat** | **κ (95% CI)** |
| 24‑hour blood pressure |  |  |  |  |
| Systolic ≥130 mm Hg |  | 251 (78·4) | 235 (73·4) | 0·44 (0·32-0·55) |
| Diastolic ≥ 80 mm Hg |  | 249 (77·8) | 244 (76·3) | 0·60 (0·50-0·71) |
| Systolic and diastolic ≥130/≥80 mm Hg |  | 208 (65·0) | 196 (61·3) | 0·52 (0·42-0·61) |
| Awake blood pressure |  |  |  |  |
| Systolic ≥135 mm Hg |  | 235 (73·4) | 227 (70·9) | 0·46 (0·35-0·56) |
| Diastolic ≥85 mm Hg |  | 215 (67·2) | 202 (63·1) | 0·53 (0·43-0·62) |
| Systolic and diastolic ≥135/85 mm Hg |  | 183 (57·2) | 164 (51·3) | 0·48 (0·38-0·58) |
| Asleep blood pressure |  |  |  |  |
| Systolic ≥120 mm Hg |  | 230 (71·9) | 215 (67·2) | 0·52 (0·42-0·62) |
| Diastolic ≥70 mm Hg |  | 275 (85·9) | 277 (86·6) | 0·47 (0·33-0·61) |
| Systolic and diastolic ≥120/≥70 mm Hg |  | 213 (66·6) | 199 (62·2) | 0·52 (0·43-0·62) |

Values are number (%) of patients among 320 patients randomised in ANTI-MASK. κ denotes the kappa statistics for the concordance in the classification of ambulatory hypertension. Meaning of the k statistic: <0 less than chance agreement; 0·01-0·20 slight agreement; 0·21-0·40 fair agreement; 0·41-0·60 moderate agreement; 0·61-0·80 substantial agreement; 0·81-0·99 perfect agreement.

**Table S2:** **Comparison of patients randomised and not randomised**

| **Characteristic** | **Randomised**  **(n = 320)** | **Not randomised**  **(n = 109)** | **p** |
| --- | --- | --- | --- |
| No. with characteristics (%) |  |  |  |
| Women | 138 (43·1) | 58 (53·2) | 0·068 |
| Men | 182 (56·9) | 51 (46·8) |
| Current smoking | 65 (20·3) | 12 (11·0) | 0·029 |
| Drinking alcohol | 71 (22·2) | 19 (17·4) | 0·29 |
| Diabetes* | 14 (4·4) | 1 (0·9) | 0·13 |
| Previous antihypertensive treatment‡ | 49 (15·3) | 17 (15·6) | 0·94 |
| Mean (SD) of measurements |  |  |  |
| Age, y | 54·0 (9·7) | 52·0 (8·9) | 0·12 |
| Body mass index, kg/m^2^§ | 24·4 (2·7) | 24·4 (2·9) | 0·88 |
| Office systolic blood pressure, mm Hg | 130·2 (6·7) | 128·6 (11·1) | 0·15 |
| Office diastolic blood pressure, mm Hg | 81·4 (6·3) | 83·3 (7·7) | 0·024 |
| 24‑hour systolic blood pressure, mm Hg | 136·9 (9·4) | 132·6 (11·7) | 0·0009 |
| 24‑hour diastolic blood pressure, mm Hg | 84·7 (6·5) | 84·0 (6·4) | 0·32 |
| Serum creatinine, µmol/L** | 74·5 (15·5) | 75·5 (13·7) | 0·78 |
| eGFR, mL/min/1.73 m^2^¶** | 92·5 (12·9) | 94·8 (10·4) | 0·45 |
| Total serum cholesterol, mmol/L** | 5·19 (1·06) | 5·39 (1·22) | 0·41 |
| HDL serum cholesterol, mmol/L#** | 1·31 (0·34) | 1·42 (0·28) | 0·17 |
| Fasting plasma glucose, mmol/L** | 5·34 (0·83) | 5·12 (0·40) | 0·037 |
| No. with target organ damage (%) |  |  |  |
| Left ventricular hypertrophy& | 25 (7·8) | 2 (1·8) | 0·023 |
| baPWV ≥1400 cm/s¦ | 312 (97·5) | 33 (30·3) | <0·0001 |
| Microalbuminuria‖ | 38 (11·9) | 4 (3·7) | 0·014 |

Patients declining consent are excluded from the patients screened but not randomised.

* Diabetes is a diagnosis documented in hospital records, using antidiabetic drugs or a fasting plasma glucose of ≥ 7 mmol/L.

‡ Previous antihypertensive treatment was discontinued for ≥2 weeks prior to screening.

§ Body mass index is body weight in kilogram divided by body height in meters squared.

¶ eGFR is the glomerular filtration rate estimated from serum creatinine by the Chronic Kidney Disease Epidemiology Collaboration formula.

# HDL indicates high-density lipoprotein.

& Left ventricular hypertrophy is a Sokolow-Lyon index of ≥3·5 mV in women and ≥4·0 mV in men or Cornell product ≥2440 mm × ms.

¦ baPWV indicates brachial-ankle pulse wave velocity.

ǁ Microalbuminuria is a baseline urinary albumin-to-creatinine ratio (ACR) of ≥3·5 mg/mmol in women and ≥2·5 mg/mmol in men. Baseline level was the ACR of the first mid-morning urine samples obtained at the screening visits.

** Conversion factors: creatinine from mmol/L to mg/dL, multiply by 0·0113; eGFR from mL/min/1·73m2 to mL/s/1·73m2, multiply 0·0167; cholesterol from mmol/L to mg/dL, multiply by 38·67; glucose from mmol/L to mg/dL, multiply by 18·02.

**Table S4: Number of ambulatory blood pressure readings**

| **Characteristic** | **n** | **Median** | **P25-P75** | **P5-P95** | **Range** |
| --- | --- | --- | --- | --- | --- |
| **Screening visit 1** |  |  |  |  |  |
| 24-hour blood pressure | 320 | 61 | 58-63 | 51-65 | 34-67 |
| Daytime blood pressure | 320 | 43 | 40-46 | 32-50 | 19-57 |
| Nighttime blood pressure | 320 | 16 | 14-18 | 11-22 | 6-30 |
| **Screening visit 2** |  |  |  |  |  |
| 24-hour blood pressure | 320 | 62 | 58-64 | 51-65 | 42-78 |
| Daytime blood pressure | 320 | 44 | 40-47 | 32-52 | 24-61 |
| Nighttime blood pressure | 320 | 16 | 14-18 | 11-23 | 3-33 |
| **At 2 months** |  |  |  |  |  |
| 24-hour blood pressure | 283 | 61 | 57-63 | 47-65 | 39-76 |
| Daytime blood pressure | 283 | 44 | 39-47 | 30-51 | 21-59 |
| Nighttime blood pressure | 283 | 16 | 14-17 | 11-20 | 7-33 |
| **At 4 months** |  |  |  |  |  |
| 24-h hour blood pressure | 215 | 62 | 57-64 | 50-65 | 36-79 |
| Daytime blood pressure | 215 | 44 | 39-47 | 32-51 | 21-65 |
| Nighttime blood pressure | 215 | 15 | 14-17 | 10-21 | 2-24 |
| **At 12 months** |  |  |  |  |  |
| 24-hour blood pressure | 250 | 61 | 57-63 | 47-65 | 38-66 |
| Daytime blood pressure | 250 | 43 | 39-46 | 30-50 | 23-54 |
| Nighttime blood pressure | 250 | 15 | 14-17 | 11-20 | 0-30 |

N indicates the number of patients with an ambulatory blood pressure recording.

**Table S5: Blood pressure in the** **secondary intention-to-treat analysis**

| **Blood pressure** | **Active treatment  (n = 153)** | **Placebo  (n = 167)** | **Between-group difference** | **p** |
| --- | --- | --- | --- | --- |
| Office SBP, mm Hg |  |  |  |  |
| Baseline | 130·4 (5·4) | 129·4 (6·4) | 1·0 (-0·3 to 2·3） |  |
| Adjusted changes | -8·8 (0·9)‡ | 0·1 (0·9) | -8·9 (-11·3 to -6·5) | <0·0001 |
| Office DBP, mm Hg |  |  |  |  |
| Baseline | 81·7 (5·2) | 81·2 (6·4) | 0·5 (-0·8 to 1·7) |  |
| Adjusted changes | -4·2 (0·6)‡ | 0·9 (0·6) | -5·2 (-6·8 to -3·5) | <0·0001 |
| 24‑hour SBP, mm Hg |  |  |  |  |
| Baseline | 136·2 (7·7) | 136·5 (9·4) | -0·3 (-2·2 to 1·6) |  |
| Adjusted changes | -10·0 (0·9)‡ | -1·4 (0·9) | -8·6 (-11·0 to -6·2) | <0·0001 |
| 24-hour DBP, mm Hg |  |  |  |  |
| Baseline | 84·5 (5·7) | 84·4 (6·5) | 0·1 (-1·3 to 1·4) |  |
| Adjusted changes | -6·4 (0·5)‡ | -1·1 (0·5)* | -5·3 (-6·6 to -4·0) | <0·0001 |
| Daytime SBP, mm Hg |  |  |  |  |
| Baseline | 140·2 (8·3) | 140·9 (10·4) | -0·7 (-2·7 to 1·4) |  |
| Adjusted changes | -10·2 (1·0)‡ | -1·7 (0·9) | -8·5 (-11·2 to -5·9) | <0·0001 |
| Daytime DBP, mm Hg |  |  |  |  |
| Baseline | 87·2 (6·2) | 87·5 (7·3) | -0·3 (-1·8 to 1·2) |  |
| Adjusted changes | -6·5 (0·5)‡ | -1·4 (0·5)† | -5·1 (-6·5 to -3·7) | <0·0001 |
| Nighttime SBP, mm Hg |  |  |  |  |
| Baseline | 125·8 (10·9) | 125·6 (10·3) | 0·3 (-2·1 to 2·6) |  |
| Adjusted changes | -9·3 (1·0)‡ | -1·0 (0·9) | -8·3 (-11·0 to -5·7) | <0·0001 |
| Nighttime DBP, mm Hg |  |  |  |  |
| Baseline | 77·5 (6·6) | 76·7 (6·7) | 0·8 (-0·6 to 2·3) |  |
| Adjusted changes | -6·2 (0·6)‡ | -1·1 (0·6) | -5·1 (-6·7 to -3·5) | <0·0001 |

Baseline blood pressure levels are mean (SD). Adjusted changes are mean (SE) derived by mixed models based on observed data. Models include randomisation group as class variable and account for the baseline blood pressure level, sex and age as fixed effects and patient as random effect. Between-group differences (active treatment minus placebo) are presented as mean (95% confidence interval). p refers to the significance of the between-group difference. Baseline office and ambulatory blood pressure are entered in the models as averages of the measurements obtained at the two screening visits. SBP=systolic blood pressure, DBP=diastolic blood pressure. Daytime and nighttime are the awake and asleep periods of the day as recorded in the patient diaries. Significance of the within-group change: * p<0·05; † p<0·01; ‡ p<0·001.

**Table S6:** **Target organ damage in the secondary intention-to-treat analysis**

| **Variables** | **Active treatment  (n = 153)** | **Placebo  (n = 167)** | **Between-group  difference** | **p** |
| --- | --- | --- | --- | --- |
| Sokolow-Lyon index, mV |  |  |  |  |
| Baseline | 2·08 (0·65) | 2·06 (0·73) | 0·01 (-0·14 to 0·17) |  |
| Adjusted changes | -0·12 (0·04)† | 0·02 (0·04) | -0·13 (-0·23 to -0·04) | 0·0080 |
| Cornell product, mm × ms |  |  |  |  |
| Baseline | 1416·4 (505·5) | 1515·2 (652·8) | -98·8 (-228·1 to 30·4) |  |
| Adjusted changes | -80·2 (27·4)† | 15·1 (27·2) | -95·3 (-170·8 to -19·7) | 0·014 |
| baPWV, cm/s |  |  |  |  |
| Baseline | 1622·1 (180·9) | 1610·0 (199·7) | 12·1 (-30·1 to 54·3) |  |
| Adjusted changes | -153·7 (14·9)‡ | -27·7 (14·6) | -125·9 (-166·8 to -85·1) | <0·0001 |
| ACR, mg/mmol |  |  |  |  |
| Baseline | 1·27 (0·79-1·97) | 1·20 (0·67-1·90) | 0·06 (-0·17 to 0·35) |  |
| Adjusted changes | -0·17 (0·09)* | 0·16 (0·08) | -0·29 (-0·45 to -0·10) | 0·0039 |

Baseline values are mean (SD) or geometric mean (interquartile range). Adjusted changes are mean (SE) derived by mixed models based on observed data. Models include randomisation group as class variable and account for the baseline value of the variable, sex and age as fixed effects and patient as random effect. Between-group differences (active treatment minus placebo) are presented as mean (95% confidence interval). p refers to the significance of the between-group difference. Baseline values of the urinary albumin-to-creatinine ratio are averages of the measurements obtained at the two screening visits 1‑month apart. baPWV=brachial-ankle pulse wave velocity, ACR=urinary albumin-to-creatinine ratio. Significance of the within-group change: * p<0·05; † p<0·001; ‡ p<0·001.

**Table S7: Quality-of-life score in the** **primary intention-to-treat analysis**

| **Variables** | **Active Treatment  (n = 153)** | **Placebo  (n = 167)** | **Between-group  difference** | **p** |
| --- | --- | --- | --- | --- |
| Physical health |  |  |  |  |
| Baseline | 65·2 (11·3) | 67·8 (11·7) | -2·6 (-5·1 to -0·0） |  |
| Adjusted changes | 0·1 (0·8) | 0·6 (0·8) | -0·4 (-2·7 to 1·9) | 0·73 |
| Psychological |  |  |  |  |
| Baseline | 61·2 (13·3) | 64·4 (12·4) | -3·2 (-6·0 to -0·3) |  |
| Adjusted changes | 0·2 (1·0) | 1·3 (0·9) | -1·1 (-3·8 to 1·6) | 0·42 |
| Social relationships |  |  |  |  |
| Baseline | 65·0 (12·9) | 68·3 (13·3) | -3·3 (-6·2 to -0·4) |  |
| Adjusted changes | 0·5 (1·0) | 0·4 (1·0) | 0·0 (-2·7 to 2·8) | 0·98 |
| Environment |  |  |  |  |
| Baseline | 59·5 (12·3) | 61·7 (12·9) | -2·2 (-5·0 to 0·5) |  |
| Adjusted changes | 1·1 (0·9) | 2·4 (0·9)* | -1·2 (-3·7 to 1·2) | 0·33 |

Quality of life was scored in four domains, using the brief investigator-administered questionnaire developed by the World Health Organization (WHOQOL-BREF). Domain scores are transformed to 0-100 scale. Baseline values are mean (SD). Adjusted changes are least square means (SE). In the primary ITT analysis, missing values are substituted by multiple imputation (see Statistical Methods). Mixed models include randomisation group as class variable and account for the baseline value of the variable, sex and age as fixed effects and patient as random effect. Between-group differences are presented as mean (95% confidence interval). p refers to the significance of the between-group difference. Significance of the within-group change: *p<0·01.

**Table S8:** **Patients not included in the per-protocol analysis**

| **Reason** | **Active treatment** | **Placebo** |
| --- | --- | --- |
| **Patients exiting the trial or withdrawn*** | **29** | **39** |
| Lost to follow-up | 12 | 13 |
| Withdrawal of consent | 11 | 13 |
| Uncontrolled hypertension | 2 | 8 |
| Minor symptoms | 4 | 3 |
| Medical event leading to withdrawal† | 0 | 2 |
| **Patients violating the protocol** |  |  |
| ***Withdrawn from the trial**** | **7** | **10** |
| Age <30 or >70 y at inclusion | 2 | 1 |
| Screening BP outside protocol range | 1 | 0 |
| Screening ambulatory BP missing | 1 | 0 |
| No TOD at screening visit | 1 | 1 |
| Exclusion criteria met at screening | 1 | 1 |
| Did not use allocated drugs | 0 | 1 |
| Use of open-label antihypertensive drugs | 0 | 3 |
| Missed drug titration at follow-up 2/4 | 1 | 3 |
| ***Completing the 12‑month follow-up***‡ | **7** | **5** |
| Age <30 or >70 y at inclusion | 1 | 1 |
| Screening BP outside protocol range | 0 | 1 |
| Exclusion criteria met at screening | 1 | 0 |
| Missed drug titration at follow-up 2/4 | 5 | 3 |
| Total excluded from the per-protocol analysis§ | **36** | **44** |

* Of the patients who were withdrawn, 7 randomised to active treatment and 10 randomised to placebo did not meet the eligibility criteria or deviated from the standardised protocol regulating the prescription of study medication.

† Two serious events leading to withdrawal of the patient by the physician occurred in the placebo group: one patient diagnosed with prostate cancer and one with cholelithiasis.

‡ Patients randomised and followed up for 12 months, but not meeting the eligibility criteria or deviating from the standardised protocol regulating the prescription of study medication.

§ The number of patients included in the per-protocol analysis amounted to 117/153 (76·5%) randomised to active treatment and 123/167 (73·7%) randomised to placebo.

BP=blood pressure; TOD=target organ damage.

**Table S9****:** **Blood pressure in the per-protocol analysis**

| **Blood pressure** | **Active treatment  (n = 117)** | **Placebo  (n = 123)** | **Between-group difference** | **p** |
| --- | --- | --- | --- | --- |
| Office SBP, mm Hg |  |  |  |  |
| Baseline | 130·1 (5·4) | 129·3 (6·0) | 0·7 (-0·7 to 2·2) |  |
| Adjusted changes | -8·8 (0·9)‡ | -0·1 (0·9) | -8·7 (-11·2 to -6.2) | <0·0001 |
| Office DBP, mm Hg |  |  |  |  |
| Baseline | 81·6 (5·1) | 81·5 (6·0) | 0·05 (-1·4 to 1·5) |  |
| Adjusted changes | -4·5 (0·6)‡ | 0·8 (0·6) | -5·3 (-7·0 to -3·6) | <0·0001 |
| 24‑h SBP, mm Hg |  |  |  |  |
| Baseline | 136·1 (7·7) | 137·0 (9·5) | -0·9 (-3·1 to -1·3) |  |
| Adjusted changes | -10·1 (1·0)‡ | -1·6 (0·9) | -8·4 (-11·0 to -5·9) | <0·0001 |
| 24-h DBP, mm Hg |  |  |  |  |
| Baseline | 84·6 (5·7) | 85·1 (6·4) | -0·5 (-2·0 to 1·1) |  |
| Adjusted changes | -6·6 (0·5)‡ | -1·2 (0·5)* | -5·4 (-6·8 to -4·0) | <0·0001 |
| Daytime SBP, mm Hg |  |  |  |  |
| Baseline | 140·2 (8·4) | 141·4 (10·3) | -1·1 (-3·5 to 1·3) |  |
| Adjusted changes | -10·1 (1·0)‡ | -1·9 (1·0) | -8·2 (-10·9 to -5·5) | <0·0001 |
| Daytime DBP, mm Hg |  |  |  |  |
| Baseline | 87·5 (6·2) | 88·2 (7·2) | -0·7 (-2·4 to 1·0) |  |
| Adjusted changes | -6·5 (0·5)‡ | -1·4 (0·5)† | -5·2 (-6·6 to -3·7) | <0·0001 |
| Nighttime SBP, mm Hg |  |  |  |  |
| Baseline | 125·2 (11·0) | 126·1 (10·6) | -1·0 (-3·7 to 1·8) |  |
| Adjusted changes | -9·7 (1·0)‡ | -1·1 (1·0) | -8·6 (-11·3 to -5·8) | <0·0001 |
| Nighttime DBP, mm Hg |  |  |  |  |
| Baseline | 77·2 (6·7) | 77·6 (6·6) | -0·4 (-2·1 to 1·3) |  |
| Adjusted changes | -6·5 (0·6)‡ | -1·0 (0·6) | -5·4 (-7·1 to -3·8) | <0·0001 |

Baseline blood pressure levels are mean (SD). Adjusted changes are least square means (SE) derived by mixed models based on data retained the per-protocol analysis (see methods). Models include randomisation group as class variable and account for the baseline blood pressure level, sex and age as fixed effects and patient as random effect. Between-group differences are presented as mean (95% confidence interval). p refers to the significance of the between-group difference. Baseline office and ambulatory blood pressure are averages of the measurements obtained at the two screening visits. SBP=systolic blood pressure, DBP=diastolic blood pressure. Daytime and nighttime are the awake and asleep periods of the day as recorded in the patient diaries. Significance of the within-group change: * p<0·05; † p<0·01; ‡ p<0·001.

**Table S10:** **Target organ damage in the per-protocol analysis**

| **Variables** | **Active treatment  (n = 117)** | **Placebo  (n = 123)** | **Between-group  difference** | **p** |
| --- | --- | --- | --- | --- |
| Sokolow-Lyon index, mV |  |  |  |  |
| Baseline | 2·06 (0·64) | 2·07 (0·76) | -0·01 (-0·19 to 0·17) |  |
| Adjusted changes | -0·10 (0·04)† | 0·03 (0·04) | -0·14 (-0·24 to -0·33) | 0·0094 |
| Cornell product, mm × ms |  |  |  |  |
| Baseline | 1409·2 (508·5) | 1502·1 (652·2) | -92·9 (-242·5 to 56·7) |  |
| Adjusted changes | -82·9 (29·0)† | 11·4 (28·3) | -94·3 (-173·6 to -15·0) | 0·020 |
| baPWV, cm/s |  |  |  |  |
| Baseline | 1612·3 (169·5) | 1606·9 (170·9) | 5·4 (-37·9 to 48·7) |  |
| Adjusted changes | -150·8 (15·8)‡ | -24·0 (15·2) | -126·8 (-169·7 to -83·9) | <0·0001 |
| ACR, mg/mmol |  |  |  |  |
| Baseline | 1·20 (0·79-1·73) | 1·21 (0·66-1·86) | -0·01 (-0·26 to 0·30) |  |
| Adjusted changes | -0·10 (0·08) | 0·25 (0·08)† | -0·28 (-0·44 to -0·10) | 0·0041 |

Baseline values are mean (SD) or geometric mean (interquartile range). Adjusted changes are least square means (SE) derived by mixed models based on data retained in the per-protocol analysis (see methods). Models include randomisation group as class variable and account for the baseline value of the variable, sex and age as fixed effects and patient as random effect. Between-group differences are presented as mean (95% confidence interval). p refers to the significance of the between-group difference. Baseline values of the urinary albumin-to-creatinine ratio are analysed as averages of the measurements obtained at the two screening visits. baPWV=brachial-ankle pulse wave velocity, ACR=urinary albumin-to-creatinine ratio. Significance of the within-group change: † p<0·01; ‡ p<0·001.

| **Table S11:** **Safety analysis by treatment group** | | | | |
| --- | --- | --- | --- | --- |
| **Variables** | **Active treatment**  **(n = 150)** | | **Placebo**  **(n = 163)** | **p** |
| No. of adverse events (%) | | | | |
| Headache | | 1 (0·7) | 5 (3·1) | 0·22 |
| Chest discomfort | | 5 (3·3) | 4 (2·5) | 0·74 |
| Gastrointestinal complication | | 5 (3·3) | 4 (2·5) | 0·74 |
| Dizziness | | 3 (2·0) | 7 (4·3) | 0·34 |
| Skin itching | | 0 | 2 (1·2) | 0·50 |
| Erectile dysfunction | | 1 (0·7) | 0 | 0·48 |
| Conjunctivitis | | 0 | 1 (0·6) | >0·99 |
| No. of serious adverse events (%) † | | | | |
| Hospitalisation | | 3 (2·0) | 4 (2·5) | >0·99 |
| Tumour | | 2 (1·3) | 2 (1·2) | >0·99 |
| Cerebral infarction | | 1 (0·7) | 0 | 0·48 |
| Macula hole | | 0 | 1 (0·6) | >0·99 |
| Cholelithiasis | | 0 | 1 (0·6) | >0·99 |
| No. of incident biochemical abnormalities (%) | | | | |
| Fasting plasma glucose ≥7·0 mmol/L | | 2 (1·3) | 3 (1·8) | >0·99 |
| ALT >40 IU/L | | 7 (4·7) | 10 (6·1) | 0·57 |
| AST >40 IU/L | | 5 (3·3) | 2 (1·2) | 0·27 |
| No. of incident abnormal ECG signs (%) | | | | |
| Sinus rhythm <60 beats/min | | 1 (0·7) | 2 (1·2) | >0·99 |
| sinus rhythm >100 beats/min | | 1 (0·7) | 2 (1·2) | >0.99 |
| ST-T change | | 8 (5·3) | 5 (3·1) | 0·40 |
| Atrial premature beats | | 2 (1·3) | 0 | 0·23 |
| Ventricular premature beats | | 1 (0·7) | 1 (0·6) | >0.99 |
| No. of patients with any event (%) | | 38 (25·3) | 43 (26·4) | 0·83 |

† Serious adverse events occurred were those required a hospitalisation. No patient died during this trial. The p values are derived by Fisher exact test. Because patients could experience multiple adverse events, the numbers of events in this table do not add up to the number of patients. Abbreviations: ALT=alanine aminotransferase; AST=aspartate aminotransferase; ECG=electrocardiogram.


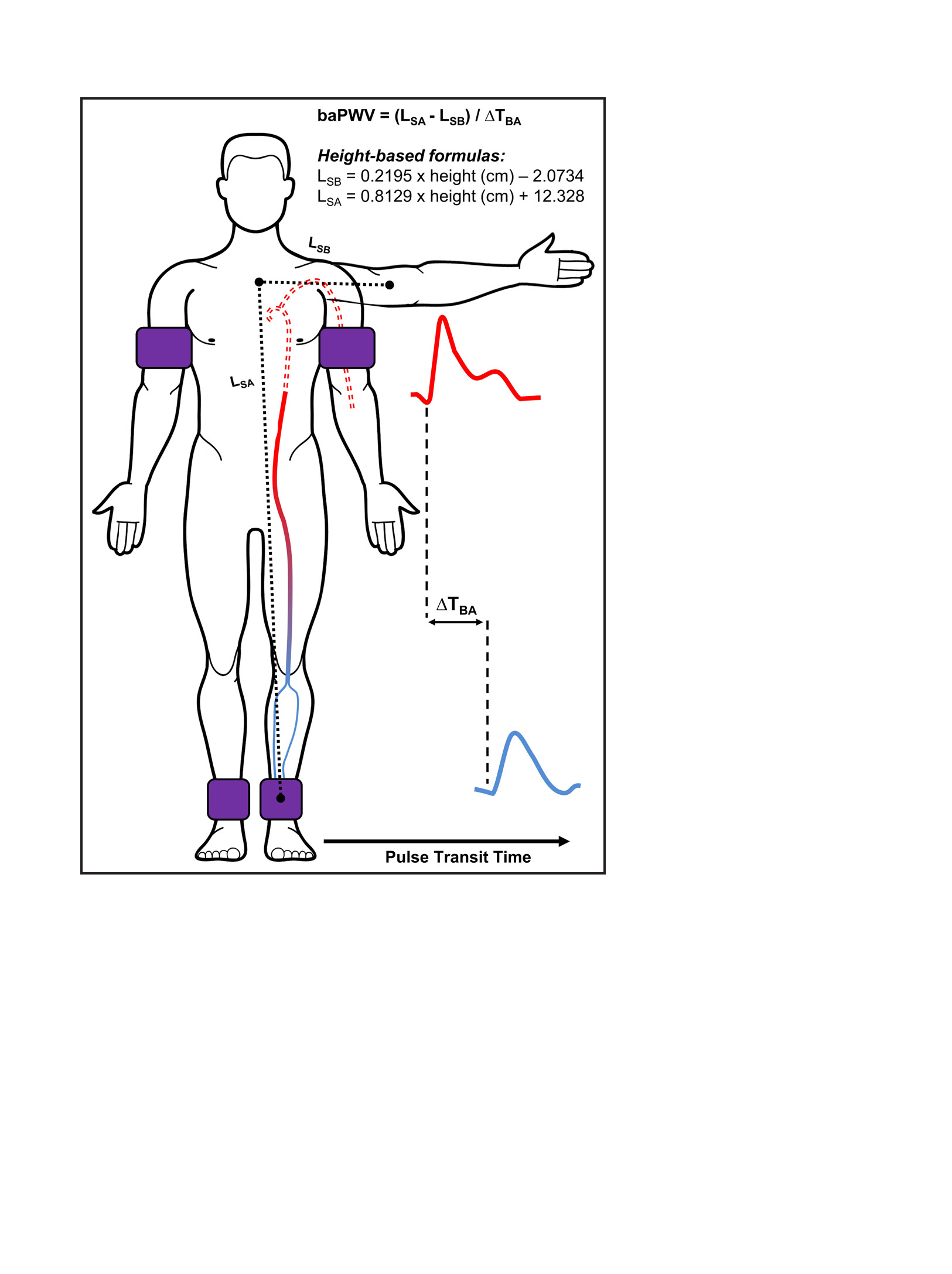


***Figure S1:***

**Measurement of brachial-ankle pulse wave velocity**baPWV is estimated as the length between the sternal notch and the ankle (LSA) minus the sternal notch to brachium LSB), as determined by body surface measurements or height-based formulas, divided by the time delay between the foot of the brachial and ankle waveforms (ΔTBA). Reproduced with permission from Stone K et al. Hypertension 2023;80:1980-1992. (doi: 10.1161/HYPERTENSIONAHA.123.21314).


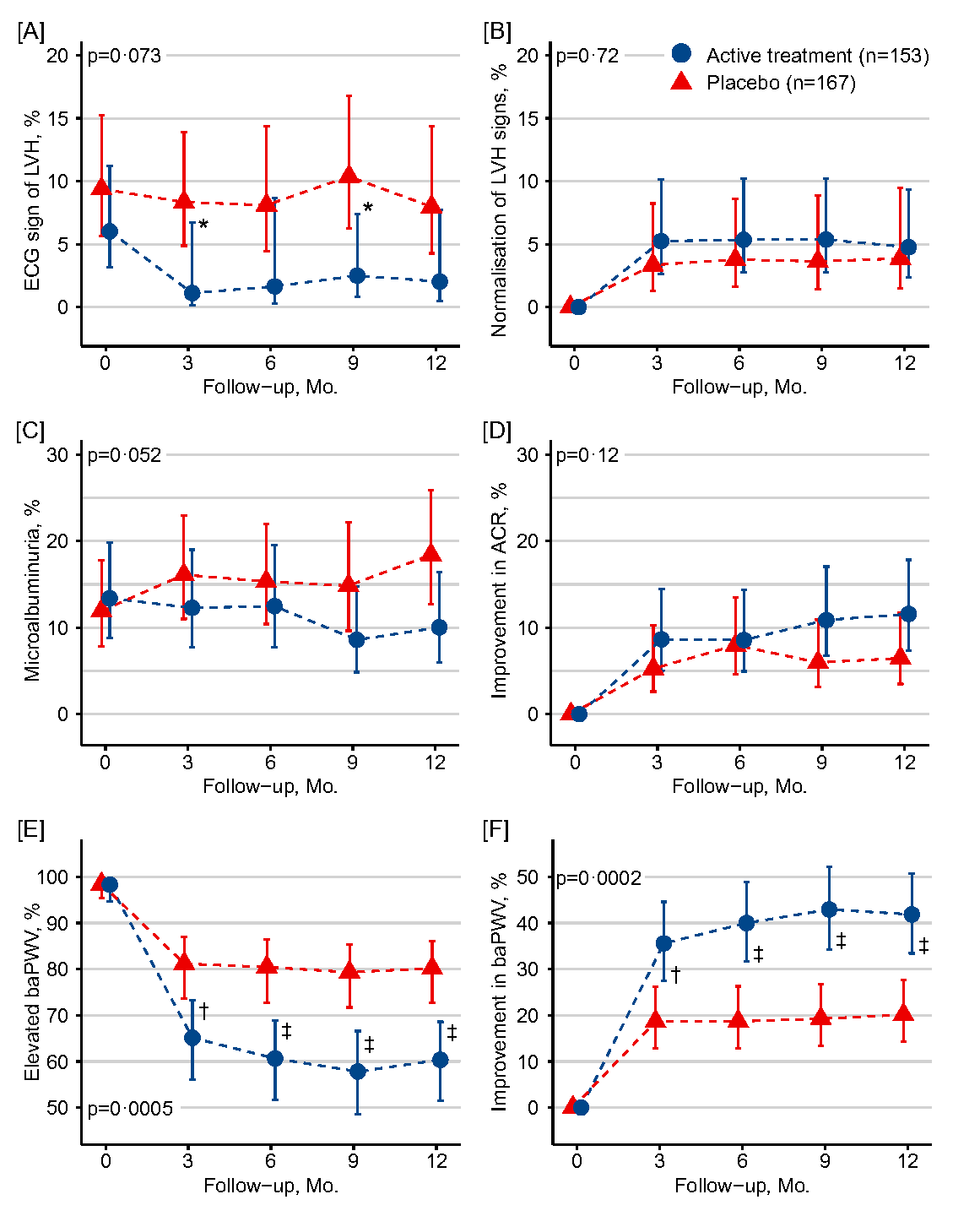


***Figure S2:***

**Individual TOD component in the primary intention-to-treat analysis**The figure shows the time trends by randomisation group for the prevalence of individual TOD component and its regression rate. p-values indicate the significance of the between-group difference over all time points combined. Symbols indicate the within-group differences with baseline as reference: * p<0·01, † p<0·001, ‡ p<0·001. TOD=target organ damage, LVH= Left ventricular hypertrophy, ACR= urinary albumin-to-creatinine ratio, baPWV=brachial-ankle pulse wave velocity.


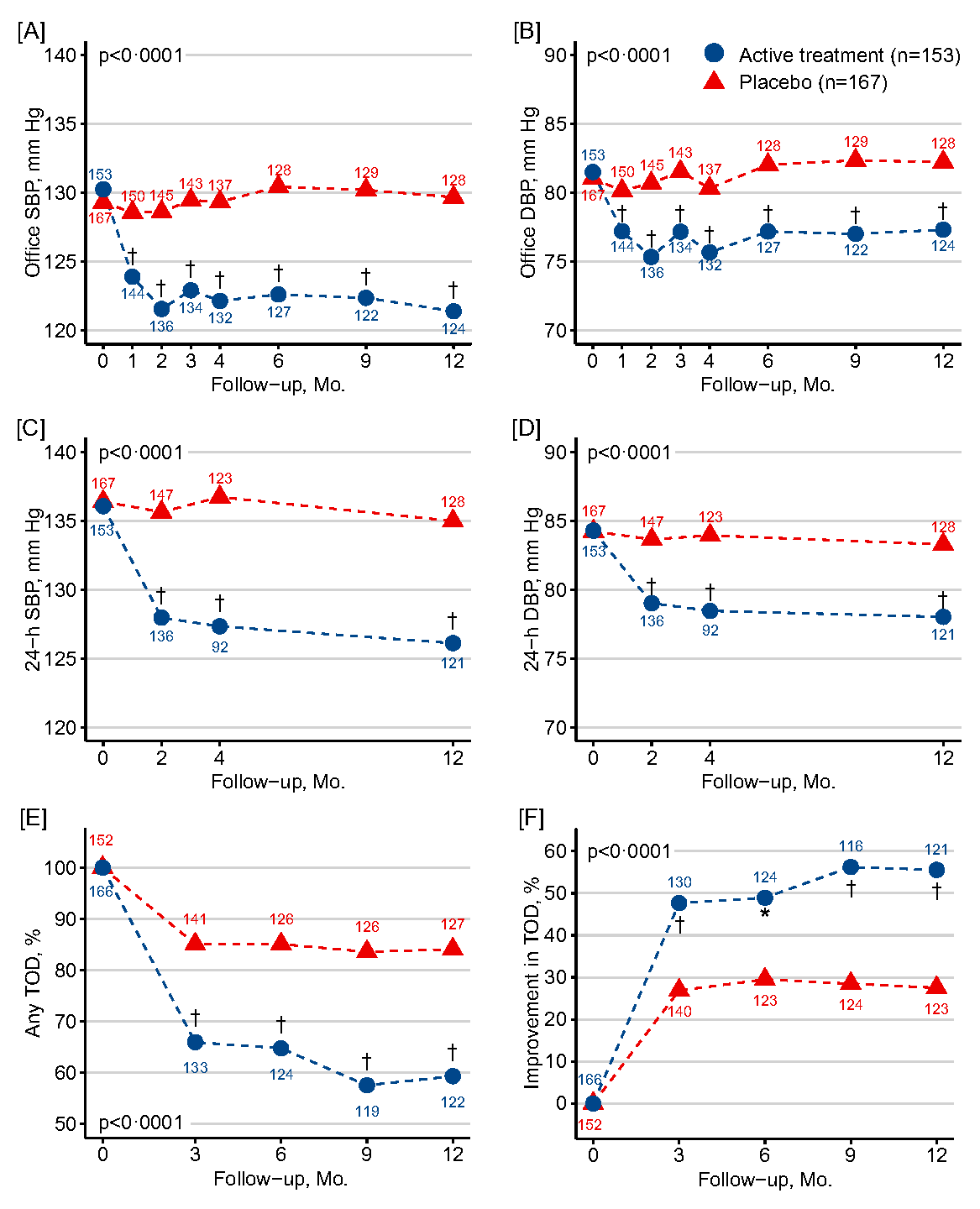


***Figure S3:***

**Blood pressure and target organ damage in the secondary intention-to-treat analysis** The figure shows the time trends by randomisation group for systolic and diastolic office blood pressure (panels A and B), 24-hour systolic and diastolic ambulatory blood pressure (panels C and D), the prevalence of any form of target organ damage (panel E), and the regression rate of target organ damage, the primary study outcome (panel F). Numbers adjacent to the plotted points indicate the number of patients with measurements. p-values indicate the significance of the between-group difference over all time points combined. Symbols indicate the within-group differences with baseline as reference: * p<0·01, † p<0·001. SBP=systolic blood pressure, DBP=diastolic blood pressure, TOD=target organ damage.


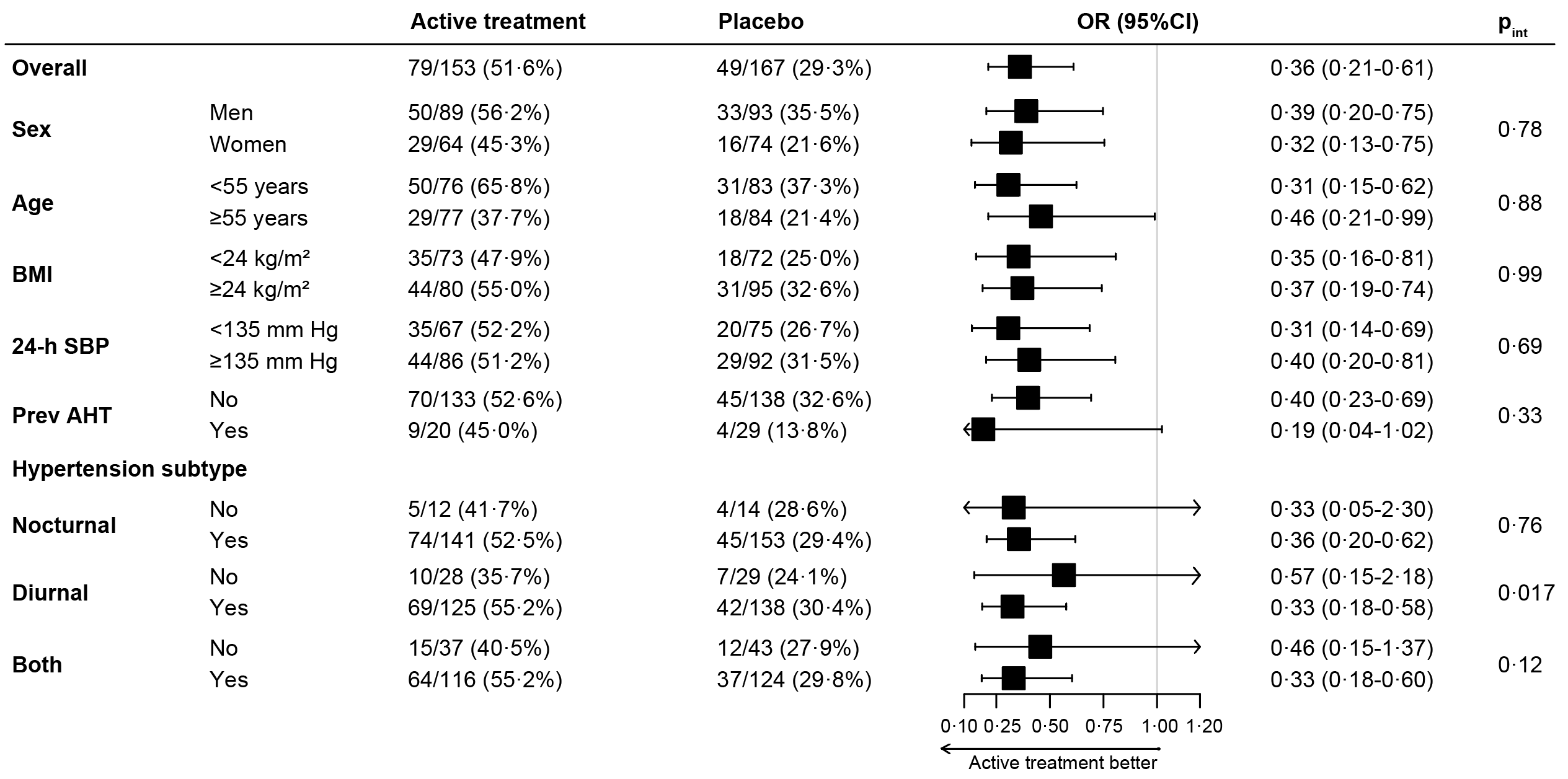


***Figure S4:***

**Improvement of target organ damage by subgroups**

The primary ITT analysis is stratified according to sex, medians of age, body mass index, and 24-hour systolic blood pressure at baseline, history of antihypertensive treatment prior to screening, and the presence versus absence of 24‑hour, daytime or nighttime hypertension. Ambulatory blood pressure levels are means of two screening visits. Odds ratios (OR) given with 95% confidence interval contrast active treatment to placebo. ORs are derived by generalised estimating equations with repeated measures and are adjusted for sex and age, if these variables are not used for stratification. Pint indicates the significance of the subgroup-by-randomisation interaction. Squares represent the point estimate of the ORs and horizontal lines the 95% confidence interval. BMI=body mass index, 24‑h SBP=24-hour systolic blood pressure, Prev AHT= previous antihypertensive treatment.


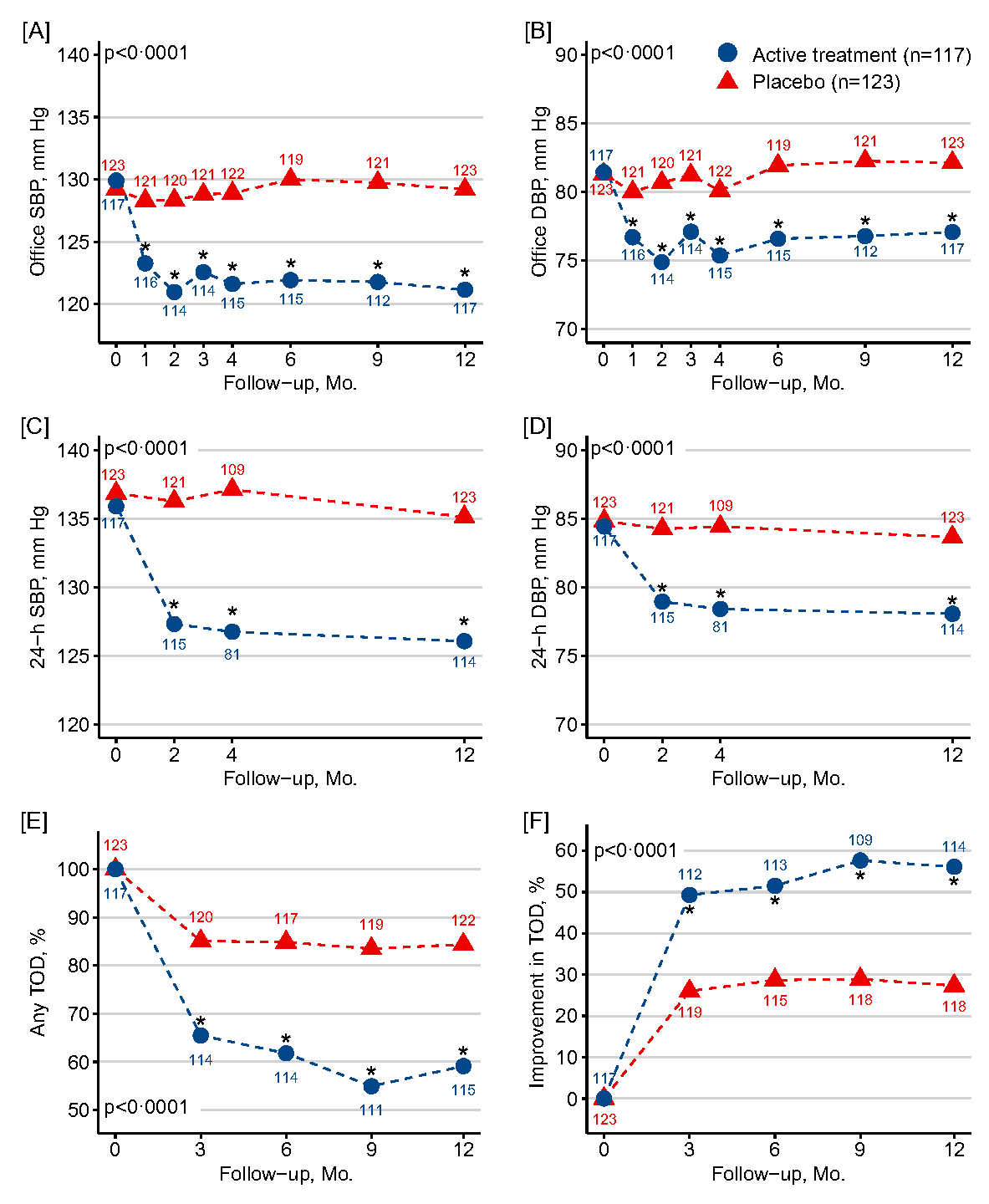


***Figure S5:***

**Blood pressure and target organ damage in the per-protocol analysis**The figure shows the time trends by randomisation group for systolic and diastolic office blood pressure (panels A and B), the 24-hour systolic and diastolic ambulatory blood pressure (panels C and D), the prevalence of any form of target organ damage (panel E), and the regression rate of target organ damage, the primary study outcome (panel F). p-values indicate the significance of the between-group difference over all time points combined. Numbers adjacent to the plotted points indicate the number of patients with measurements. Symbols indicate the within-group differences with baseline as reference: * p<0·001. SBP=systolic blood pressure, DBP=diastolic blood pressure, TOD=target organ damage.

**CLINICAL TRIAL PROTOCOL**

**Antihypertensive Treatment of Masked Hypertension
for Target Organ Protection (ANTI-MASK)**

**A Multicenter Randomized Double-Blind Placebo-Controlled Trial**

**Clinicaltrials.gov Identifier:** **NCT 02893358**

**Version 2.0**

**Version date: June 17, 2020**

**PROTOCOL VERSION AND AMENDMENT TRACKING**

| Version Number | Protocol Date | Ethics Approval Date | Amendment |
| --- | --- | --- | --- |
| Version 1.0 | July 10, 2016 | Oct 8, 2016 | -- |
| Version 2.0 | June 17, 2020 | July 14, 2020 | The total sample size increased from 300 to 320 patients for a drop-out rate of 25% instead of 10%. |

TABLE OF CONTENTS

[Synopsis 22](#__RefHeading___Toc157690811)

[1. Background 26](#__RefHeading___Toc157690812)

[2. Objectives 28](#__RefHeading___Toc157690813)

[3. Study design 28](#__RefHeading___Toc157690814)

[4. Outcome variables 30](#__RefHeading___Toc157690815)

[4.1 Primary outcome variable 30](#__RefHeading___Toc157690816)

[4.2 Secondary outcome variables 30](#__RefHeading___Toc157690817)

[4.3 Safety variables 31](#__RefHeading___Toc157690818)

[5. Trial participants 31](#__RefHeading___Toc157690819)

[5.1 Inclusion criteria 31](#__RefHeading___Toc157690820)

[5.2 Exclusion criteria 32](#__RefHeading___Toc157690821)

[5.3 Withdrawal 33](#__RefHeading___Toc157690822)

[6. Randomization and treatment 34](#__RefHeading___Toc157690823)

[7. Follow up 37](#__RefHeading___Toc157690824)

[7.1 Follow-up visits 37](#__RefHeading___Toc157690825)

[7.2 Study measurements 39](#__RefHeading___Toc157690826)

[8. Data management 45](#__RefHeading___Toc157690827)

[8.1 Data transmission and management 45](#__RefHeading___Toc157690828)

[8.2 Sample size estimation 45](#__RefHeading___Toc157690829)

[8.3 Randomization 45](#__RefHeading___Toc157690830)

[8.4 Blinding 46](#__RefHeading___Toc157690831)

[8.5 Statistical analysis 47](#__RefHeading___Toc157690832)

[9. Ethical and legal issues 47](#__RefHeading___Toc157690833)

[9.1 Risks and benefits 48](#__RefHeading___Toc157690834)

[10. Timelines 49](#__RefHeading___Toc157690835)

[11. Organization and implementation 49](#__RefHeading___Toc157690836)

[References 51](#__RefHeading___Toc157690837)

# Synopsis

**Title:** Antihypertensive Treatment in Masked Hypertension for Target Organ Protection (ANTI-MASK)

**Background:** Hypertension is a leading risk factor for cardiovascular morbidity and mortality. Masked hypertension is characterized by normal office blood pressure (BP) in the presence of elevated daytime, nighttime or 24-hour BP. Its prevalence rate was about 15-20% in untreated population and 30% in patients on antihypertensive treatment. Masked hypertension was associated with target organ damage (TOD) and increased cardiovascular risk, similar to that with sustained hypertension. It indicated that patients with masked hypertension might need antihypertensive treatment to protect target organs and reduce cardiovascular risk. However, up to now there is no direct interventional evidence showing that antihypertensive treatment can be beneficial to patients with masked hypertension.

**Objectives****:** To compare the improvement rate of TODs after 12 months of treatment between the active antihypertensive treatment and placebo in patients with masked hypertension and at least one sign of the three TODs, including left ventricular hypertrophy (LVH) diagnosed by electrocardiogram (ECG), large arterial stiffness assessed as an increased brachial-ankle pulse wave velocity (baPWV), or microalbuminuria. The improvement of TODs is defined as the normalization of any of the three TODs or of at least 20% reduction in baPWV or urinary albumin-to-creatinine ratio (ACR). Secondary observation variables include reductions in the 24-hour, daytime, and nighttime systolic/diastolic BPs after 2 and 12 months of the active versus placebo treatment, the reductions in ECG indices of LVH (Cornell product and Sokolow-Lyon index), ACR, and baPWV, and the incidence of all-cause mortality and cardiovascular events (stroke, myocardial infarction) within one year.

**Study Design:** This study is a multicenter, randomized, double-blind, placebo-controlled clinical trial with two same-sized treatment groups: active antihypertensive treatment (based on allisartan) and placebo treatment.

**Study Participants:** Patients with masked hypertension and at least one sign of TODs, aged 30 to 70 years, will be eligible to the study. Patients who have not been treated for hypertension or are off treatment for at least 2 weeks, and have at least one sign of the following TODs can be screened: ECG diagnosed LVH (Cornell product ≥2440 mm × ms or Sokolow-Lyon index ≥3.5 mV in women and ≥4.0 mV in men), large arterial stiffness (baPWV ≥1400 cm/s), or microalbuminuria (ACR of ≥3.5 mg/mmol in women and ≥2.5 mg/mmol in men in each of the two mid-morning urine samples collected on different screening days). The 24-h ambulatory BP monitoring will be performed at screening visit 1. If a patient has a 24-hour, daytime and nighttime ambulatory BP of ≥130/80 mm Hg, or ≥135/85 mm Hg, and ≥120/70 mm Hg respectively, the patient will be invited to perform office and ambulatory BP and urinary ACR measurements within 4 weeks. If the patient’s office BP remains <140/90 mm Hg, and the 24-h BP ≥130/80 mm Hg or daytime BP ≥135/85 mm Hg or nighttime BP ≥120/70 mm Hg, and one sign of TOD can be confirmed after examinations, the patient can be randomly assigned to the active or placebo treatment. The exclusion criteria include: the patient is on antihypertensive drug treatment, with suspicious or confirmed secondary hypertension, sleep apnea, diabetic nephropathy, renal parenchymal diseases such as chronic nephritis or polycystic kidney diseases, liver dysfunction (serum alanine transaminase [ALT], aspartate aminotransferase [AST], or total bilirubin [TBL] above 2 times of normal limits), serum creatinine concentration ≥2.0 mg/dL (176 µmol/L), serum potassium ≥5.5 mmol/L), onset of cardiovascular diseases within recent 6 months, such as coronary heart disease, myocardial infarction, stroke, hypertrophic or dilated cardiomyopathy, or contraindications of the angiotensin receptor blockers.

**Randomization and Treatment:** After stratification for center, sex and the presence versus absence of nighttime hypertension, eligible patients will be randomized in a 1:1 proportion to antihypertensive drug treatment (allisartan 80 mg per day) or matching placebo. If the ambulatory BP is uncontrolled (i.e. 24-h BP ≥130/80 mm Hg or daytime BP ≥135/85 mm Hg or nighttime BP ≥120/70 mm Hg), the allisartan dose should be doubled to 160 mg once daily from month 2 onwards and amlodipine 2.5 mg once daily should be added at the 4-month visit. In the control group, matching placebos are used likewise. It is recommended to take the drugs at 8 to 9 o’clock in the morning daily.

**Baseline and Follow-up:**  All eligible patients should sign an informed consent form approved by the ethics committee before randomization. At baseline, medical history will be inquired, and clinical examinations including the 24-h ambulatory BP monitoring, ECG, baPWV, blood and urine biochemistry tests will be performed. Follow-up visits are scheduled at monthly intervals until month 4 and at 3-month intervals from month 6 to 12. At each follow-up visit, office BP measurement will be performed, study drugs will be collected and dispensed, and adverse events will be inquired. The 24-h ambulatory BP monitoring should be repeated at 2- and 12-months follow-up. For those with uncontrolled ambulatory BP at 2 months follow-up, the 24-h ambulatory BP monitoring will be repeated again at 4-month visit. The assessment of TOD (ECG, baPWV, and urinary ACR) will be repeated at 3, 6, 9, and 12 months of follow-up. After 12 months of treatment, blood and urine routine tests and biochemical tests will also be repeated.

**Sample Size and Statistical Analysis:** The primary outcome is the difference in the improvement rate of TODs between the active treatment group and placebo group at 12 months of follow-up. Assuming a 40% of the improvement rate in the active treatment group and a 20% of the improvement rate in the placebo group, α= 0.05, with a 90% of power and a 25% of dropout rate, 160 eligible patients per group and thus a total of 320 patients will be needed. The SAS software will be used for data management and analysis. The test will be used for the between-group comparisons of categorical data.

**Timelines:** Patients will be recruited from Sep 2016 to Sep 2020, and follow-up will be completed in Sep 2021.

**Organization and Implementation:** The study will be coordinated by the Shanghai Institute of Hypertension. The principal investigators are Professor Yan Li and Professor Ji-Guang Wang. It is planned to invite 15 to 20 hospitals to participate in the trial; it is expected that 10 to 30 patients can be randomized at each hospital.

# 1. Background

A large body of research underscored that hypertension is the leading risk factor for cerebrovascular and cardiovascular morbidity and mortality [1]. Compared to office blood pressure (BP), ambulatory BP is more closely associated with the incidence of cardiovascular and cerebrovascular disease [2]. Masked hypertension, characterized by normal office BP in the presence of an elevated 24-h, daytime or nighttime ambulatory BPs, is an important subtype of hypertension, which requires 24-h ambulatory BP monitoring for diagnosis. Its prevalence is approximately 15-20% among untreated individuals and 30% among patients on antihypertensive treatment [3]. Domestic and international studies have shown that measures of target organ damage (TOD), such as left ventricular mass index, carotid intima-media thickness, and the urinary microalbumin concentration, increase in patients with masked hypertension to a similar extent as in patients with sustained hypertension [4]. Outcome studies consistently revealed that patients with masked hypertension, irrespective of treatment status, had an increased cardiovascular risk compared to normotensive individuals [5]. Patients with masked hypertension, either isolated daytime, isolated nighttime or day-night hypertension, have a nearly two-fold increased incidence risk of fatal or non-fatal cardiovascular events compared to those with normal BP [6]. This evidence suggests the that antihypertensive drug treatment might be required in in patients with masked hypertension to mitigate the risk of TOD and cardiovascular complications. However, to date, there is no direct clinical trial evidence supporting that antihypertensive treatment will be beneficial to patients with masked hypertension.

Angiotensin II (Ang II), the active octapeptide component of the renin-angiotensin-aldosterone system (RAS) and plays a pivotal role in the development of cardiovascular diseases, particularly hypertension. Angiotensin II receptor blockers (ARBs) competitively inhibit Ang II from binding to angiotensin II type 1 (AT1) receptors, thereby counteracting vasoconstriction, sympathetic stimulation, and the aldosterone-stimulating effects of Ang II. Since the approval of the first ARB, losartan, for clinical use in 1994, several other ARBs, such as telmisartan, irbesartan, and olmesartan, have been introduced in the market. Globally conducted clinical trials consistently demonstrated that ARBs not only reduced BP but also reversed myocardial hypertrophy, enhanced left ventricular function, decreased urinary microalbumin excretion, improved kidney function, and lowered the risk of cardiovascular events [7-8]. ARBs are therefore particularly appropriate to the hypertensive patients with TOD, such as left ventricular hypertrophy (LVH), heart failure, diabetic nephropathy, microalbuminuria, or proteinuria, with minimal adverse effects.

Allisartan is the first novel ARB independently developed by China. It be metabolized by esterase extensively in the gastrointestinal tract to EXP3174, which is identical to the metabolite of losartan via the hepatic CYP450 pathway. EXP3174 can selectively bind to the AT1 receptors, blocking the pathological effects of Ang II [9]. Preclinical, toxicity, and clinical trial studies consistently showed that allisartan can effectively lower BP with excellent safety and few adverse events [10-12]. A noteworthy advantage of allisartan lies in its metabolic pathway of gastrointestinal enzymatic hydrolysis, eliminating the need for hepatic catabolism, thereby reducing potential drug interactions. In clinical trials, allisartan effectively and safely reduced BP, and achieved high control rates in patients with mild to moderate hypertension [13]. Hence, we choose allisartan as the first-line active antihypertensive drug in this multicenter, randomized, double-blind, placebo-controlled clinical trial with as objective to explore whether antihypertensive treatment improves TOD in patients with masked hypertension.

# 2. Objectives

The primary objective of this study is to compare the improvement rate of TOD by 12 months of antihypertensive treatment versus placebo in patients with masked hypertension and at least one sign of TOD. The three signs of TOD considered are LVH diagnosed by electrocardiogram (ECG), stiffness of the large arteries manifested as an increase in brachial-ankle pulse wave velocity (baPWV), and microalbuminuria. Improvement of TOD is defined as the regression of any of the three signs of TOD to the normal range or a decrease by at least 20% in baPWV or the urinary albumin-to-creatinine ratio (ACR). The secondary endpoints include the between-group differences of the following measures: the 24-h, daytime, and nighttime systolic/diastolic BP after treatment for 2 and 12 months; decreases in ECG voltages (Sokolow-Lyon index and Cornell product), urinary ACR, and baPWV after treatment for 12 months; the improvement in quality of life score [14] after 12 months of treatment; and the incidence of all-cause mortality and cardiovascular events (myocardial infarction, stroke) within 12 months of randomization.

# 3. Study design

This study is a multicenter, randomized, double-blind, placebo-controlled clinical trial with two treatment groups of 160 patients each: the active antihypertensive treatment group (based on allisartan) and the placebo group (based on matching placebo). The study flowchart is as follows:


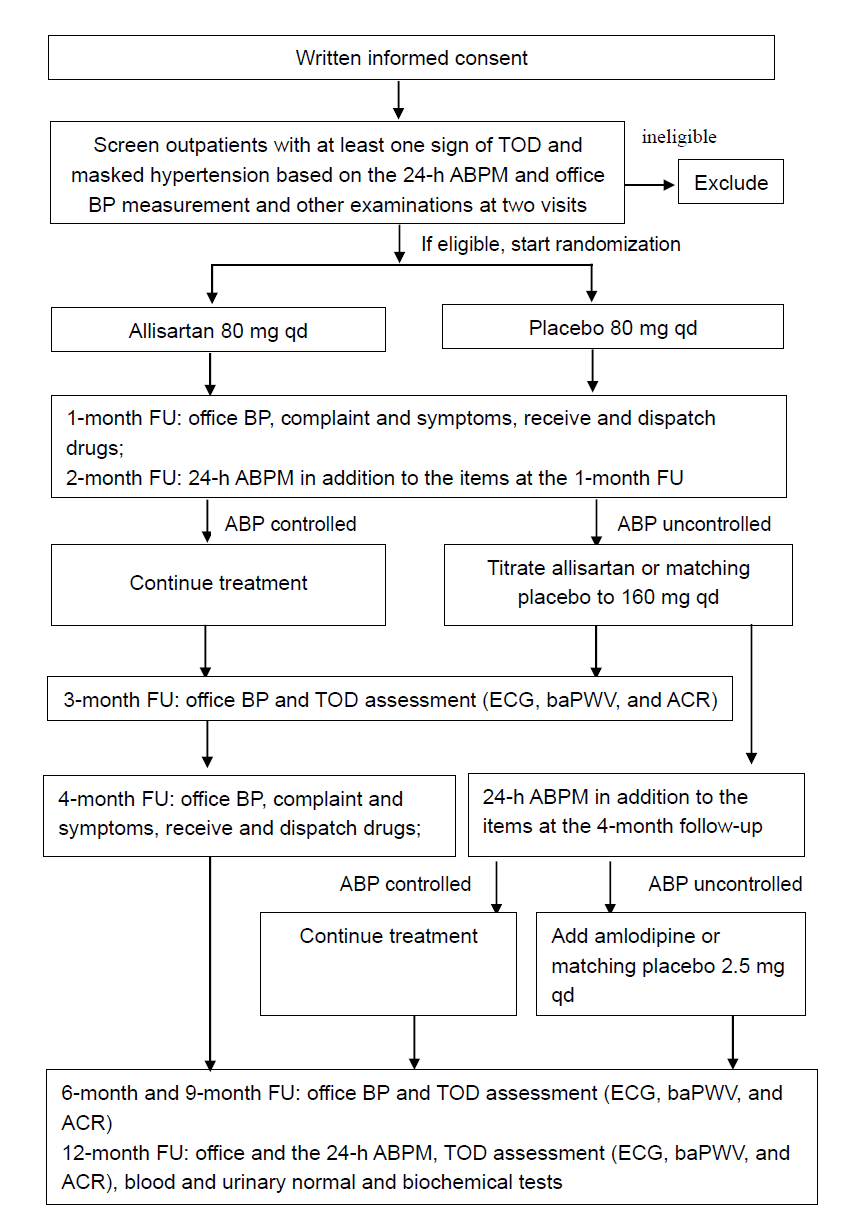


Fig.1 Study flow chart.

Abbreviations: ABPM, ambulatory blood pressure monitoring; ACR, albumin-to-creatinine ratio; baPWV, brachial-ankle pulse wave velocity; BP, blood pressure; ECG, electrocardiogram; FU, follow-up; TOD, target organ damage.

# 4. Outcome variables

## 4.1 Primary outcome variable

The primary outcome variable is the difference in the improvement rate of TOD after 12 months of active antihypertensive treatment versus placebo in patients with masked hypertension and at least one sign of TOD (ECG-diagnosed LVH, arterial stiffness presenting as an increase in baPWV, or microalbuminuria). The improvement of TOD is defined as the normalization of any of the three signs of TOD or the reduction by at least 20% in baPWV or ACR.

## 4.2 Secondary outcome variables

The secondary outcome variables include the between-group (active versus placebo treatment) differences in the following measures:

1. Reductions in the 24-h, daytime, and nighttime systolic/diastolic BPs after 2- and 12-months treatment. Daytime and nighttime intervals are defined according to the awake or sleeping time recorded in the patients’ diary or short-clock fixed time intervals. According to the short-clock time intervals, daytime and nighttime are the intervals from 8:00 to 20:00 h and from 23:00-5:00 h, respectively.
2. The reductions in ECG indexes of LVH (Sokolow-Lyon index and Cornell product), ACR, and baPWV after 3, 6, 9, and 12 months of treatment.
3. The incidence of all-cause mortality and cardiovascular events (myocardial infarction, stroke) within 12 months of randomization.
4. The between-group difference in the quality-of-life score (WHO-QOL-BRIEF [14]) after 12 months of treatment.

## 4.3 Safety variables

All adverse events (AE) and serious adverse events (SAE) that occur during the study must be recorded and reported. An adverse event is defined as any adverse medical event observed during the conduct of the trial. A serious adverse event is defined as any adverse event that results in death, life-threatening complications, hospitalization, permanent or severe disability or incapacity, or is considered as an important medical event by the investigator. For any AE or SAE, the investigator must evaluate and report its possible relation to the study drug. All AE and SAE should be reported by the investigator in the case report form. If the occurrence of SAE is possibly related to the study drug, the investigator must report it to the study sponsor within 24 hours after being aware of the event. Suspected unexpected serious adverse reactions (SUSARs) will be reported to the national regulatory authorities and relevant ethics committees by the study sponsor. SUSARs will also be communicated to all investigators. In addition to AEs, SAEs and SUSARs, patient safety will be monitored by vital signs, and routine laboratory blood and urinary tests.

# 5. Trial participants

This study will enroll approximately 320 eligible hypertensive patients (randomized into two equal treatment groups with 160 patients per group).

## 5.1 Inclusion criteria

1. Male or female patient;
2. Age of 30-70 years old;
3. Patient has never been on antihypertensive treatment or stopped taking blood pressure lowering drugs for at least 2 weeks;
4. Patient has masked hypertension at each of the two screening visits 4 weeks apart, defined as office BP (average of 3 readings at each visit) <140/90 mm Hg, while the 24-h ambulatory BP is ≥130/80 mm Hg and (or) the daytime (awake) BP is ≥135/85 mm Hg and (or) the nighttime (sleeping) BP is ≥120/70 mm Hg;
5. Patient has at least one sign of TOD: ECG-diagnosed LVH (Cornell product ≥2440 mm × ms or Sokolow-Lyon index ≥3.5 mV in women and ≥4.0 mV in men), stiffness of the large arteries (baPWV ≥1400 cm/s), or microalbuminuria (ACR of ≥3.5 mg/mmol in women and ≥2.5 mg/mmol in men in each of the two mid-morning urine samples collected on different screening days);
6. Patient is willing to participate in the trial and able to come to hospital to be followed up;
7. Patient is mentally competent and willing to sign the informed consent form.

## 5.2 Exclusion criteria

1. The 24-h ABPM does not include the required number of error-code free readings (<70% of the programmed readings, or number of BP readings during the awake/daytime period <20 or during the sleeping/nighttime period <7);
2. The patient is currently on antihypertensive treatment;
3. Suspected or confirmed secondary hypertension;
4. Suspected or confirmed sleep apnea syndrome;
5. Has to take medications which may influence BP, such as alpha-blockers for prostate disease;
6. Diabetes with microalbuminuria;
7. Renal parenchymal disease, such as chronic nephritis, polycystic kidney and etc.;
8. Unstable coronary heart disease, myocardial infarction, heart failure or stroke within 6 months before being considered for randomization;
9. Structural heart diseases, such as valvular heart disease, congenital heart disease, hypertrophic cardiomyopathy, or dilated cardiomyopathy;
10. Previous or current atrial fibrillation or frequent arrhythmia;
11. Previous history of peripheral arterial disease, such as arteritis or aneurysm;
12. Contraindications for ARB treatment (hypersensitivity reaction to any ARB, history of angioedema, significant bilateral renal artery stenosis or single functional kidney, mid- and late-term pregnancy, lactation, hemodynamic-related renal artery stenosis, hypotension or hemodynamic instability);
13. Abnormal liver and kidney function: serum alanine transaminase (ALT), aspartate transaminase (AST), or total bilirubin 2 times above the normal limit; serum creatinine ≥2.0 mg/dL (176 µmol/L), or serum potassium ≥5.5 mmol/L;
14. Mental illness;
15. History of malignant cancer;
16. Other serious medical conditions that the investigators consider unsuitable for participation in this study;
17. Possible poor compliance as judged by the investigator.

## 5.3 Withdrawal

Withdrawal means that the participants terminate the clinical trial for any reason.

Participants may withdraw from the study for the following reasons:

1. Participants requests to withdraw;
2. Office BP during the follow-up is ≥140/90 mm Hg at 3 visits on different days, and therefore office hypertension can be diagnosed;
3. Investigators consider that continuing the trial can be harmful to the patient;
4. The ambulatory BP recording is of insufficient quality and the participant is unwilling to repeat ABPM;
5. Medication adherence is poor (pill count indicates <80% of expected pills taken at 3 visits);
6. Patients take other drugs that may affect BP or the antihypertensive medications are changed without doctor’s authorization;
7. Unblinding of patient or doctor;
8. SAE or other events requiring emergency treatment;
9. Double-blind treatment prematurely ends or the patients is lost to follow-up or passes away;

The reason for any withdrawal from the trial must be recorded in the case report form. The investigator should carefully record the reason why the patient is defaulting from the trial and indicate whether the reason for defaulting is related to the trial procedures. Similarly, for participants withdrawing consent, the reason for their withdrawal should be recorded.

# 6. Randomization and treatment

Eligible participants will be randomly assigned into the two treatment groups, i.e., the active treatment group and the placebo group, after stratification by center, sex, and the presence of nocturnal hypertension, using a computerized random function and permuted blocks. Within each center, there are 4 patients in each block. Nocturnal hypertension is a sleeping/nighttime BP of ≥120 mm Hg systolic or ≥70 mm Hg diastolic at the second screening visit.

Treatment in the two randomized groups is as following:

In the active treatment group, patients have to take allisartan 80 mg daily orally, and in the placebo group, the placebo matching allisartan, 80 mg. At the 2-month follow-up, the 24-h ABPM will be repeated. If the ambulatory BP is uncontrolled (24-h BP ≥130/80 mm Hg, daytime BP ≥135/85 mm Hg, or nighttime BP ≥120/70 mm Hg), the dose of the drugs (allisartan or the matching placebo) will be doubled to 160 mg daily. For those with uncontrolled ambulatory BP at the 2-month follow up, the 24-h ABPM will be repeated at the 4-month follow-up. If the ambulatory BP is still uncontrolled, amlodipine 2.5 mg and matching placebo will be added for patients in the active and placebo treatment groups, respectively. In both groups, the drug treatment should be continued until the 12-month follow-up visit. Study drugs are recommended to be taken once a day at 8 to 9 o’clock in the morning. If office BP is ≥140/90 mm Hg at 3 follow-up visits on different days, the participants can be withdrawn from the trial and treated with any necessary open-label antihypertensive drug instead of the double-blind medication.

Allisartan and its placebo are produced by Shenzhen Salubris Pharmaceutical Co., Ltd. Amlodipine and its placebo are produced by Suzhou Dongrui Pharmaceutical Co., Ltd. The drug boxes will have a standardized label including a unique identification number, the number of pills inside the box, indications, usage and dosage, storage conditions, drug dispatching unit, abbreviated name of the participant, and the words "*For clinical research use only*". Each participating center should have a person in charge of the trial drugs. The drugs should be dispensed according to the drug box identification number. The person who is in charge of the trial drug at each center should fill in the "Trial Drug Dispense and Reclaim Form" in a timely manner.

**Table 1. Schedule of the trial procedures**

| Procedures | Screening visit 1 | Screening visit 2 | Randomization | Follow-up 1 | Follow-up 2 | Follow-up 3 | Follow-up 4 | Follow-up 5 | Follow-up 6 | Follow-up 7 | Unscheduled Follow-up |
| --- | --- | --- | --- | --- | --- | --- | --- | --- | --- | --- | --- |
|  | -4w | -3-0d | 0w | 4w | 8w | 12w | 16w | 24w | 36w | 48w |  |
| Informed consent | X |  |  |  |  |  |  |  |  |  |  |
| Check inclusion and exclusion criteria | X | X | X |  |  |  |  |  |  |  |  |
| Complaint and symptoms | X | X |  | X | X | X | X | X | X | X | X |
| Physical examination | X | X |  |  |  |  |  |  |  | X | X |
| Office BP measurement | X | X |  | X | X | X | X | X | X | X | X |
| 24-h ABPM | X | X |  |  | X |  | X* |  |  | X | X |
| STOP-Bang questionnaire | X |  |  |  |  |  |  |  |  |  |  |
| Blood biochemical tests | X |  |  |  |  |  |  |  |  | X | X |
| Urinary ACR | X | X |  |  |  | X |  | X | X | X | X |
| Electrocardiogram | X |  |  |  |  | X |  | X | X | X | X |
| Pulse wave velocity | X |  |  |  |  | X |  | X | X | X | X |
| Scores of quality of life |  | X |  |  |  |  |  |  |  | X | X |
| Dispense drugs |  |  | X | X | X | X | X | X | X |  |  |
| Reclaim drugs |  |  |  | X | X | X | X | X | X | X | X |

Abbreviations: ABPM, ambulatory blood pressure monitoring; ACR, albumin-to-creatinine ratio; BP, blood pressure; d, days; w, week

*Patients who have uncontrolled ambulatory BPs at the follow-up 2 should perform the 24-h ABPM at the follow-up 4.

# 7. Follow up

## Follow-up visits

The follow-up after randomization will last for 12 months. The following visits will be conducted during the study (Table 1):

1. Screening visit 1 (-4 weeks): Participants have a normal office BP without taking antihypertensive drugs and have signs of TOD. The patients will complete the STOP-BANG questionnaire [15] to rule out sleep apnea syndrome and perform examinations including the 24-h ABPM, ECG, baPWV, urinary ACR, and routine blood and urine tests.
2. Screening visit 2 (-3 to 0 days): Participants will undergo a second office BP measurement and 24-h ABPM, and the second urinary ACR test. If the diagnosis of masked hypertension can be confirmed, and the patient has at least one sign of TOD, the patient is eligible for randomization provided that all inclusion criteria are fulfilled and none of the exclusion criteria is present. In premenopausal women, a pregnancy test should be performed.
3. Randomization visit (0 week): Eligible patients will be invited to complete all required examinations, such as height and weight measurements and the quality-of-life assessment. They will then be randomized and the study drugs will be dispensed according to the random assignment.
4. Follow-up visit 1: This follow-up visit will be conducted after 1 month of treatment since randomization (4 weeks ± 3 days). Office BP will be measured and symptoms and complaints will be recorded. Unused study drugs will be reclaimed and new study drugs for the next month will be dispensed.
5. Follow-up visit 2: This follow-up visit will be conducted after 2 months of treatment since randomization (8 weeks ± 3 days). Office BP will be measured and symptoms and complaints will be recorded. Unused study drugs will be reclaimed and new study drugs for the next month will be dispensed. If necessary, the antihypertensive study medication will be adjusted according to the trial protocol.
6. Follow-up visit 3: This follow-up visit will be conducted after 3 months of treatment since randomization (12 weeks ± 3 days). Visit 3 includes measurements of office BP, ECG voltages, baPWV and urinary ACR. Symptoms and complaints will be recorded. Study drugs will be reclaimed and dispensed.
7. Follow-up visit 4: This follow-up visit will be conducted after 4 months of treatment since randomization (16 weeks ± 3 days). Office BP will be measured and symptoms and complaints will be recorded. Study drugs will be reclaimed and dispensed. For patients with uncontrolled ambulatory BP at follow-up visit 2, the 24-h ABPM will be repeated. For patients whose ambulatory BP remains uncontrolled at visit 4, the antihypertensive study medication will be adjusted according to the trial protocol.
8. Follow-up visit 5: This follow-up visit will be conducted after 6-month treatment since randomization (24 weeks ± 3 days). Visit 5 includes measurements of office BP, ECG voltages, baPWV and urinary ACR. Symptoms and complaints will be recorded. Study drugs will be reclaimed and dispensed.
9. Follow-up visit 6: This follow-up visit will be conducted after 9 months treatment since randomization (36 weeks ± 3 days). Visit 6 includes measurements of office BP, ECG voltages, baPWV and urinary ACR. Symptoms and complaints will be recorded. Study drugs will be reclaimed and dispensed.
10. Follow-up visit 7 (last follow-up visit): This follow-up and close-out visit will be conducted after 12 months of treatment since randomization (48 weeks ± 3 days). At the last study visit, measurements include office BP, the 24-h ambulatory BP, ECG voltages, baPWV, urinary ACR, routine blood and urine tests, quality-of-life assessment, and other baseline measurements, which will be repeated. Symptoms and complaints will be recorded. Unused study drugs will be reclaimed.
11. Early termination visit: If patient terminates the study before the last planned trial visit, an unscheduled visit should be performed with the same measurements as at the close-out visit (visit 7).

## 7.2 Study measurements

All eligible patients should sign the informed consent form approved by the Ethics Committee before randomization, perform 24-h ABPM, be questioned about their medical history, and undergo examinations including a physical, office BP measurement, ECG, baPWV, and routine blood and urine tests. Data will be collected at different stages of the trial as follows:

1. Clinical history taking: During follow-up visits, information on the use of medications, and symptoms and complaints should be collected, and any adverse events and the occurrence of various cardiovascular or cerebrovascular complications should be recorded.
2. Physical examination, including heart auscultation, measurements of height, body weight, and waist and hip circumference. Height and body weight will be measured using the height and weight scales in each research center after patient took off their shoes and heavy coats. Waist circumference will be measured at the level of the umbilicus and hip circumference at its maximum.
3. Office BP measurement: Office BP measurement will be performed at all visits, preferably between 7-11 AM in the morning before the patient has taken any drugs and after patients have rested seated for 5 minutes. The validated oscillometric electronic sphygmomanometer Omron HBP1100 (Omron Healthcare Co., Ltd., Kyoto, Japan) interfaced with a suitable size cuff (large cuff for arm circumference ≥32cm) will be made available to and is to be used by all clinical centers. The cuff should be snugly applied on the arm with a tight fit not allowing the observer to pass more than one finger between the cuff and the patient’s arm. Three consecutive BP readings should be obtained at intervals of 30 seconds to 1 minute. At the first visit, BP should be measured on both arms, and at each subsequent visit, BP should be measured on the arm giving the higher systolic BP values. Systolic and diastolic BP, heart rate, and the time of day at which office BP was measured should be recorded on the study forms. The average of the three BP readings will be used as the office BP for each visit.
4. 24-h ABPM: The 24-h ambulatory BP will be recorded at the two screening visits, at 2 months, at 4 months, if participants had an elevated ambulatory BP at 2 months, and at the 12‑month or study-terminating visit (if patients terminate the trial prematurely). ABPM will be performed using validated A&D TM-2430 monitors (A&D Company Ltd., Tokyo, Japan). The devices will be programmed to measure BP every 20 minutes from 6:00 to 22:00 h, and every 30 minutes from 22:00 to 6:00 h. To initiate ABPM, BP will first be manually measured once. The same arm and cuff for the office BP measurement will be chosen for 24-h ABPM. The participant should take the study drugs as usual on the day of ambulatory BP monitoring. Standardized APBM diary cards will be used to allow the participant to record his/her waking-up and go-to-sleep time and the time on the intake of medications on monitoring days. The Shuoyun ABPM Platform will be used for the initialization of the recorders and the transmission of the BP monitoring data. Qualified doctors at the Shanghai Institute of Hypertension will comment on the ABPM report. When the 24-h ABPM is of insufficient quality (number of error-free BP readings <70% of the programmed number, number of awake/daytime BP readings <20, or number of sleeping/night BP readings <7), it is recommended to repeat ABPM under the same treatment conditions. For each ambulatory BP recording, the following variables will be calculated:
   - Averages of the 24-h, daytime, nighttime, and morning BPs and heart rate. Daytime and nighttime will be defined based on the awake and sleeping time. Alternatively, for further statistical analysis, the definitions of daytime and nighttime with short-clock fixed time intervals can also be used. According to this definition, the intervals from 8:00 to 18:00 h and from 23:00 to 5:00 h are analyzed as daytime and nighttime, respectively, and the morning period as the interval from 6:00 to 10:00 h or the 2 h after waking up.
   - Variability of systolic and diastolic BPs, and heart rate in each time period, using indexes, such as the standard deviation, the weighted standard deviation, the maximum-minimum difference, average real variability (ARV), and variability independent of mean (VIM).
   - Nighttime BP fall and night-to-day BP ratio.
   - Morning surge of BP, defined as the difference between the morning BP and the average of the lowest nighttime BP and the 2 readings immediately preceding and following this trough value.
   - The ambulatory arterial stiffness index, defined as 1 minus the regression slope of the ambulatory diastolic on systolic BP.
5. Scale for the sleep apnea syndrome assessment: The STOP-BANG questionnaire [15] will be used to exclude participants suspected of sleep apnea syndrome. The STOP-BANG questionnaire in simplified Chinese involves self-administered yes/no questions in items 1-4, 6, and 8. Researchers then measure the participant's height and weight, calculate body mass index, measure neck circumference at the level of the cricoid cartilage and complete items 5 and 7 of the scale. Each item labeled "*yes*" is scored as 1, and 0 if labeled “no”. A total score of ≥5 indicates a high probability of sleep apnea and leads to screening failure.
6. ECG examination: Routine ECG examination will be performed using a calibrated 12-lead ECG device at the screening visit and at follow-up visits of 3, 5, 6, and 7. For the ease of analysis, the ECG paper speed will be uniformly set to 25 mm/s. The legend should contain at least one 1 mV calibration pulse and a time scale. Each lead should be recorded over at least 10 seconds. Artifacts caused by lead loosening should be discarded. The precordial leads should be put on the chest as follows: C1: fourth intercostal space at the right edge of the sternum; C2: fourth intercostal space at the left edge of the sternum; C3: midpoint of the line connecting C2 and C4; C4 at the intersection of the left clavicular midline and the fifth intercostal space; C5 at the same level as C4 on the left anterior axillary line; C6 at the same level as C4 on the left midaxillary line. When placing chest leads, one should ensure to position the V4-V6 leads at the same level and not along the anatomical path of the fifth intercostal space. The ECG paper data and electronic data will be transmitted to the trial coordination center after de-identification, but labeled with each patient’s unique study identification number. LVH will be defined according to the criteria of Sokolow-Lyon and Cornell product. The specific criteria are as follows:
   - Sokolow-Lyon index: SV1 + RV5 or RV6 ≥3.5 mv in women, ≥4.0 mv in men, LVH can be defined.
   - Cornell product: for men, (RaVL + SV3) × QRS interval; for women (RaVL + SV3 + 0.8 mV) × QRS interval; if the Cornell product is ≥2440 mm × ms, LVH might be present.
7. Arterial pulse wave analysis: At one of the two screening visits and follow-up visits of 3, 5, 6, and 7, four-limb BPs and baPWV will be measured with the Omron VP2000 or VP1000 device. The subject will assume the supine position in a warm, comfortable, and quiet room and rest for 5 minutes before the measurement. Experienced technicians will wrap four standard cuffs around the upper arm and around the calf just above the ankle. Larger cuffs will be selected for patients with an arm circumference of ≥32cm. Additionally, the technicians will place electrode clip on wrists to record the electrical pulses of the heart. The device measures pulse waveforms and BP through the cuff sensor, automatically calculate the time difference (∆T) of the pulse wave from the heart to the ankle and upper arms, respectively. The pulse travel distance from the sternal notch to the upper arm and from the sternal notch to the lower ankle will be calculated according to the subject's height, and finally baPWV is calculated as the pulse travel distance divided by the time difference between the transmitted pulses. At one clinic visit, the measurement will be repeated twice at a time interval of 1 minute. The second measurement of baPWV, and the average of baPWV on the left and right side will be used for statistical analysis. Arterial stiffness is defined as a baPWV ≥1400cm/s.
8. Laboratory examinations:
   - Screening visits before randomization: The mid-stream fresh random urine sample in the morning of the clinic visit will be collected for routine urine tests and the measurement of ACR. After ≥8 h of fasting, venous blood samples will be collected for routine hematological test, and tests of serum electrolytes, creatinine, uric acid, liver function (ALT\AST\AKP\γ-GT), blood glucose, blood lipids (total cholesterol, high-density lipoprotein [HDL] cholesterol, low-density lipoprotein [LDL] cholesterol, and triglycerides), etc.
   - Follow-up visit 3: Collect the mid-stream fresh morning urine for the measurement of urinary ACR.
   - Follow-up visit 5: Collect the mid-stream fresh morning urine for the measurement of urinary ACR.
   - Follow-up visit 6: Collect the mid-stream fresh morning urine for the measurement of urinary ACR.
   - Follow-up visit 7: Perform the same hematological and biochemical measurements as at the screening visit before randomization.

Urinary albumin and creatinine concentrations will be measured by immunoturbidimetry and the standard Jaffé method, respectively. Microalbuminuria is defined as a urinary ACR ≥2.5 mg/mmol in men and ≥3.5 mg/mmol in women in two mid-morning urine samples collected on different days. When ACR is used as a continuous variable for statistical analysis, the lowest value will be used for those with a urinary albumin concentration below the detection range.

1. Patient adherence to the study medication will be evaluated by pill counting at all follow-up visits. The number of pills dispensed minus the number pills returned is an estimate of the number pills taken. Adherence is expressed as the percentage of the number of pills taken relative to the number expected to be taken for any given between-visit interval. An adherence of ≥80% is considered to be good.
2. Quality of life assessment: The World Health Organization Quality of Life Brief Scale [14] will be used to assess quality of life at baseline and at the end of follow-up. The scale consists of 26 items, including two on the overall quality of life, and the remaining 24 items are divided into 4 dimensions: physical, psychological, social relationships, and environmental dimensions.

# 8. Data management

## 8.1 Data Transmission and Management

The data collected in the study, such as clinical information, results of biochemical tests, ABPM, etc. will be transferred to the case report form (CRF). Electronic data of biochemical tests and ABPM will be saved. The printed reports will be pasted in to the CRF. All data will be de-identified and sent to the study coordination center for statistical analysis. The transmitted data will be monitored and verified against the source documents at each research center by professional staff from the coordination center.

## 8.2 Sample size estimation

The main outcome variable is the difference in the improvement rate of TOD between the active antihypertensive treatment and the placebo groups at the 12-month follow-up. Assuming that the improvement rate of TOD is 40% in the active treatment group and 20% in the placebo group, α is 0.05, power of 90%, and the dropout rate is 25%, then 160 patients in each treatment group and a total of 320 patients will be required.

## 8.3 Randomization

1. Random allocation

Stratified randomization will be applied in this trial. The stratification is according to center, sex, and the presence of nocturnal hypertension (nighttime BP ≥120 mm Hg systolic or ≥70 mm Hg diastolic at the second screening visit). Within each center, there should be 4 patients in each permuted block according to sex and the presence of nocturnal hypertension. Professional staff will write a computerized random allocation program.

1. Implementation of random allocation

The active antihypertensive drugs and matching placebos with the same shape and size as the verum study medication, will be packed at the central drug dispatching center in paper boxes (allisartan verum and placebo, 31 pills per box; amlodipine verum and placebo, 28 pills per box). The staff in charge of the study drugs will run a computer program to label each box containing either active drugs or placebo with a unique random number. Finally, the boxes with active drugs or placebo will be numbered sequentially and shipped to the local drug dispatching unit at each participating center.

Research doctors in each participating hospital click the “randomization” button in the electronic CRF after checking the eligibility of the patient and input the stratification information, including sex and the status of nighttime hypertension. Computer software then immediately generates the random allocation and lists the numbers of drug boxes that should be dispensed. Doctor and patient remain unaware of any information about the random allocation.

## 8.4 Blinding

This trial is designed as double-blind. The trial participants and research doctors will not know the treatment allocation. In case of the following emergencies, blinding can be broken: (1) serious adverse events; (2) if the patient needs emergency rescue, the responsible researcher of the local center should contact the principal investigator to request unblinding. This trial will adopt a two-stage unblinding. After the blinding state has been checked and the data locked, the group information will be coded as A and B and provided to the statistician. When the statistical analysis and the summary report has been completed, the second unblinding will be performed to disclose the group allocation (active or placebo treatment) of A and B. If during the trial, blinding is lifted in >20% of patients, the double-blind character is lost.

## 8.5 Statistical analysis

Data sets of patients will be defined and analyzed as the following:

- Intention-to-treat (ITT) data set analysis: Data of all randomized patient will be analyzed, regardless of whether they completed the trial or actually received study medication or not.
- Per-protocol (PP) data set analysis: Data of patients who completed the trial and did not violate the protocol will be analyzed.

All data will be managed and analyzed using the SAS software. Between-group means and proportions will be compared by independent *t* test or Wilcoxon test, depending on the variable distribution, and the chi-square or Fisher exact test, respectively. Paired t-tests will be used to compare the within-group differences of means before and after treatment. Statistical significance is a two-tailed α-level of 0.05 or less, unless otherwise specified.

# 9. Ethical and legal issues

Clinical trials must follow the Helsinki Declaration (2008 version) and relevant regulations and laws of clinical trial research in China. Before the initiation of the trial, each center’s local Ethics Committee must approve the protocol. Before enrolling any individual, researchers must explain the purpose, procedures, and possible risks of this study to potential participants or their designated representative in written form. Patients should be informed that they have the right to withdraw from this study at any time without compromising their treatment. Before being screened, each participant must give written informed consent, using a specific form. Researchers have the responsibility to ensure that each participant has given informed consent before entering the study. The informed consent form should be kept as a clinical trial document for future reference. All participants in the study will be covered by the study insurance. All information allowing to identify the patients will be kept confidential and will not be disclosed unless in the framework of relevant laws and/or regulations, for instance in case of a medical emergency.

## 9.1 Risks and benefits

1. During the trial, if the rate of valid ambulatory BP readings is less than 70%, or the number of BP readings is less than 20 during the awake/daytime period, or less than 7 during sleep/nighttime, the 24-h ambulatory BP recording will be considered as invalid and will need to be repeated. If the repeat ambulatory BP recording is still invalid after further the patient received additional instructions, considering poor compliance and tolerance of the patient, the patient might be withdrawn from the trial.
2. Discomfort related to 24-h ABPM, such as sleep disturbance at night, skin redness and rash beneath the cuff. ABPM will be performed up to five times during the study. However, ABPM is a clinical routine examination for most hypertensive patients, the onset of related discomfort and symptoms can be reduced through education and guidance.
3. Risks related to venous blood collection (such as hematoma). During the one-year trial, blood tests need to be performed only twice, and only a small amount of blood will be required each time.
4. Intolerance to the study drugs. The antihypertensive drugs used in this trial have been widely used in clinical practice and have good tolerability. Nevertheless, it is still necessary to closely monitor possible drug-related adverse reactions. Once intolerance occurred in a patient, action needs to be taken, and if necessary, the patient should stop taking the study drug temporarily or be withdrawn from the trial. In this case, the patient can choose other types of open-label drugs for antihypertensive treatment.
5. ARBs are contraindicated in pregnant women. Therefore, pregnancy tests should be performed in all women with child-bearing potential. Women of reproductive age who are not using effective contraceptive measures should be excluded from the trial.

The direct benefits that a participant can obtain from this study include detailed health checks conducted in a specialized hypertension management center, which will be helpful to achieve the BP control and to reduce the possible risk of cardiovascular and cerebrovascular events. All examinations and evaluations conducted in the study are free of cost for the patients.

# 10. Timelines

It is planned that the clinical trial will be started at the end of September 2016, that 320 eligible patients will be enrolled and randomized before September 2020, and that follow-up will be completed before September 2021. Database construction and statistical analysis will then be done, and 1-2 papers will be published in Chinese or SCI journals in 2022.

# 11. Organization and implementation

The Center for Epidemiological Studies and Clinical Trials, the Shanghai Institute of Hypertension, Ruijin Hospital Affiliated to Shanghai Jiao Tong University School of Medicine initiated and will coordinate this study. Professor Yan Li and Professor Ji-Guang Wang are the principal investigators of this trial. It is planned to invite 15 to 20 secondary or tertiary hospitals to participate in the trial. The expectation is that each hospital will hopefully recruit 10 to 30 patients.

# References

1. Lewington S, Clarke R, Qizilbash N, et al. Age-specific relevance of usual blood pressure to vascular mortality: a meta-analysis of individual data for one million adults in 61 prospective studies. *Lancet.* 2002;360:1903-1913.
2. Mancia G, Fagard R, Narkiewicz K et al; Task Force Members. 2013 ESH/ESC Guidelines for the management of arterial hypertension: the Task Force for the management of arterial hypertension of the European Society of Hypertension (ESH) and of the European Society of Cardiology (ESC). *J Hypertens.* 2013;31:1281-1357.
3. Franklin S, O’Brien E, Thijs L, et al. Masked hypertension: A phenomenon of measurement. Hypertension. 2015;65:16-20.
4. Tomiyama M, Horio T, Yoshii M, et al. Masked hypertension and target organ damage in treated hypertensive patients. *Am J Hypertens.* 2006;19:880-886.
5. Hansen TW, Kikuya M, Thijs L, et al; IDACO Investigators. Prognostic superiority of daytime ambulatory over conventional blood pressure in four populations: a meta-analysis of 7,030 individuals. *J Hypertens.* 2007;25:1554-1564.
6. Asayama K, Thijs L, Li Y, et al; IDACO Investigators. Setting thresholds to varying blood pressure monitoring intervals differentially affects risk estimates associated with white-coat and masked hypertension in the population. *Hypertension.* 2014;64:935-942.
7. Julius S, Kjeldsen SE, Weber M, et al. Outcomes in hypertensive patients at high cardiovascular risk treated with regimens based on valsartan or amlodipine: the VALUE randomized trial. *Lancet.* 2004;363:2022-2031.
8. Kalaitzidis R, Bakris GL. Effects of angiotensin II receptor blockers on diabetic nephropathy. *J Hypertens Suppl.* 2009;27:S15-21.
9. Stearns RA, Chakravarty PK, Chen R, et al. Biotransformation of losartan to its active carboxylic acid metabolite in human liver microsomes. Role of cytochrome P4502C and 3A subfamily members. *Drug Metab Dispos.* 1995;23: 207-215.
10. Wu MY, Ma XJ, Yang C, et al. Effects of allisartan, a new AT(1) receptor blocker, on blood pressure and end-organ damage in hypertensive animals. *Acta Pharmacol Sin.* 2009;30(3):307-313.
11. Liu Y, Wang H, Cheng Y, et al. A 26-week repeated-dose toxicity study of allisartan isoproxil in Sprague-Dawley rats. *Drug Chem Toxicol.* 2013;36: 440-450.
12. Li Y, Li XH, Huang ZJ, et al. A randomized, double blind, placebo-controlled, multicenter phase II trial of Allisartan Isoproxil in essential hypertensive population at low-medium risk. *PLoS One.* 2015;10:e0117560.
13. Jing S, Sun NL, Zhang SY, et al. The efficacy and safety of allisartan in patients with mild to moderate essential hypertension. *Chin J Clin Pharm.* 2013;29:728-731.
14. The WHOQOL Group. Development of the World Health Organization WHOQOL-BREF quality of life assessment. *Psychol Med.* 1998;28:551-558.
15. Chung F, Yegneswaran B, Liao P, Chung SA, Vairavanathan S, Islam S, Khajehdehi A, Shapiro CM. STOP questionnaire: a tool to screen patients for obstructive sleep apnea. *Anesthesiology.* 2008;108:812-821.
